# Exosome-mediated transfer of bta-miR-2484 upregulates JAK-STAT pathway to inhibit BoHV-1 replication by targeting SOCS5

**DOI:** 10.1186/s13567-026-01875-y

**Published:** 2026-11-09

**Authors:** Mengen Xu, Min A, Ying Yu, Muhammad Farhan Rahim, Haofang Yuan, Chao Jin, Hongwen Zhao, Lan He, Yan Li, Jiakui Li

**Affiliations:** 1 College of Veterinary Medicine, Huazhong Agricultural University, Wuhan, 430070 China; 2 Hubei Jiangxia Laboratory, Wuhan, 430200 China; 3 Sichuan Academy of Grassland Sciences, Chengdu, 611731 China

**Keywords:** BoHV-1, exosome, miRNA, SOCS5, JAK-STAT

## Abstract

Bovine herpesvirus-1 (BoHV-1)-induced infectious bovine rhinotracheitis has had a considerable economic impact on the global cattle industry. Exosomes have recently attracted significant interest in the field of viral infections, primarily due to their capacity to facilitate the transfer of genetic material between cells. MicroRNAs (miRNAs) have been demonstrated to play a crucial role in virus–host interactions, given their status as the most abundant nucleic acid in exosomes. However, existing research on the relationship between host cell exosomes and their miRNAs and BoHV-1 replication remains limited. The present study revealed that BoHV-1 infection leads to increased exosome release, and that exosomes released from BoHV-1-infected MDBK (Madin-Darby bovine kidney) cells inhibit BoHV-1 proliferation. Furthermore, we found that BoHV-1 infection resulted in changes to the expression profile of miRNAs within exosomes. The miRNA bta-miR-2484 is one of the differentially expressed miRNAs that is significantly upregulated and targets suppressor of cytokine signaling 5 (SOCS5), leading to its downregulation. This activates the JAK-STAT pathway and promotes the expression of interferon-stimulated genes (ISG15, OAS1), boosting the antiviral response against BoHV-1. Together, these results show that BoHV-1 infection leads to the selective enrichment of bta-miR-2484 in exosomes, which then inhibit viral replication by priming JAK-STAT antiviral signaling via SOCS5. This study provides novel insights into the immunoregulatory function of exosomal miRNAs during BoHV-1 infection and highlights exosomal bta-miR-2484 as a potential therapeutic target for controlling BoHV-1-associated disease.

**Supplementary Information:**

The online version contains supplementary material available at https://doi.org/10.1186/s13567-026-01875-y.

## Introduction

Bovine herpesvirus-1 (BoHV-1) is one of the major pathogens causing bovine respiratory disease (BRD) [1]. The virus is the primarily cause of infectious bovine rhinotracheitis, and is thus also referred to as infectious bovine rhinotracheitis virus (IBRV) [2]. Furthermore, miscarriage, conjunctivitis, encephalitis, endometritis, vaginitis, and other conditions may be symptoms of the condition [3]. BoHV-1 is categorized as an α-herpesvirus, a subfamily of enveloped double-stranded DNA viruses, and is characterized by its high transmissibility [4]. The first isolation of the virus from diseased cattle was carried out by Madin in 1956, and it was identified as a herpesvirus by Huck in 1964 [5, 6]. Although infectious bovine rhinotracheitis (IBR), caused by BoHV-1 infection, has a relatively low mortality rate, it severely impairs cattle growth and milk production, resulting in significant economic losses. Consequently, IBR is designated as a notifiable disease by the World Organisation for Animal Health (formerly OIE) and is also classified as a Category II animal disease in China [7].

Extracellular vesicles (EVs), comprising both exosome and microvesicle types, represent a heterogeneous group of membrane structures released by all cells, including prokaryotic and eukaryotic organisms [8]. In contrast to microvesicles shed from the plasma membrane, exosomes originate from the endosomal system, with diameters typically ranging from 30 to 200 nm [9]. The prevailing hypothesis initially was that exosome functions were to facilitate the transport of cellular waste. However, as research progressed, it was discovered that they carry complex nucleic acids, proteins, and metabolites to other cells, thereby playing a crucial role in cellular communication [10]. The process of viral infection necessarily involves intercellular communication, whether it be the suppression of the host cell’s immune response by the virus or the immune cells’ defense reaction against viral invasion. It is evident that exosome participation in the interaction between viruses and their hosts is an inevitable occurrence [11]. It has been demonstrated that these particles can shuttle immune factors or viral structural proteins within cells, thereby activating immune pathways [12–14]. However, certain viruses have evolved novel immune evasion mechanisms that utilize exosomes to facilitate intercellular genetic material transmission [8, 15, 16].

MicroRNA (miRNA) was first discovered in 1993 by Lee et al. in the nematode *Caenorhabditis elegans* as a class of endogenous non-coding RNA molecules ranging from 20 to 24 nucleotides in length [17–19]. It has been demonstrated that certain viruses, including herpesviruses and polyomaviruses, are capable of encoding miRNAs [20, 21]. Unlike messenger RNAs (mRNAs), miRNAs do not encode proteins but instead regulate gene expression at the post-transcriptional level [22]. The primary function of these proteins is to regulate gene expression, which is achieved through their binding to complementary sites in the 3′ untranslated region (UTR) of mRNAs [23]. This regulatory process is involved in various cellular processes, including growth, proliferation, differentiation, and cell cycle regulation. In the context of pathological states, such as cancer or immune-mediated diseases, the expression profile of miRNAs is subject to corresponding alterations [24, 25]. Consequently, differentially expressed miRNAs are being widely applied in clinical settings as biomarkers for the onset of certain diseases [9, 26–28]. miRNA is the predominant form of functional RNA in exosomes, where it demonstrates stability and exerts regulatory effects [29, 30]. In certain viral infections, miRNAs within exosomes also play a crucial role. In the context of enterovirus 71 (EV71) infection, the role of the exosome is to mediate the suppression of the type I interferon response by miR-146a [16]. Herpes simplex virus-1 (HSV-1)-encoded miR-H28 and miR-H29 are also exported via exosomes, thereby enabling their latent state within ganglia [31]. Although several studies have examined the involvement of miRNAs in BoHV-1 infection, the contribution of exosome-associated miRNAs to this process remains largely unexplored.

The JAK-STAT pathway is a widely distributed intracellular signaling cascade that plays a central role in regulating inflammatory and immune responses [32]. Upon viral infection, interferon signaling activates the downstream JAK-STAT pathway. Specifically, the binding of interferon-α (IFN-α)/β to its membrane receptors, IFNAR1 and IFNAR2, initiates intracellular signal transduction, resulting in STAT activation and nuclear translocation. This process ultimately induces the transcription of interferon-stimulated genes, thereby mediating antiviral immune responses [33, 34]. The suppressor of cytokine signaling (SOCS) family, consisting of SOCS1–7 and CIS, is a key negative regulatory factor of the JAK-STAT signaling pathway [35]. SOCS proteins can effectively inhibit the activation of the JAK-STAT cascade, restrict excessive activation of cellular immune responses, and maintain immune homeostasis in vivo [36]. Growing evidence suggests that multiple viruses exploit host immune regulatory pathways by inducing SOCS protein expression, thereby dampening antiviral immune responses and facilitating viral replication and pathogenicity. In particular, influenza A virus (IAV) infection has been reported to markedly upregulate SOCS1 and SOCS3 expression, resulting in suppression of host antiviral immunity [37]. Moreover, SOCS2 knockout has been reported to attenuate BoHV-5-induced meningoencephalitis, further highlighting the important regulatory role of the SOCS family in viral infections [38]. Moreover, the SOCS protein family has been identified as a common and crucial target for miRNA-mediated post-transcriptional regulation in various inflammatory and infectious diseases. miR-122 can target SOCS1 to modulate virus-induced lung disease, and miR-155-5p exerts a regulatory effect on the progression of pulmonary arterial hypertension by targeting SOCS5 [39, 40].

In the present study, we demonstrated that BoHV-1 infection resulted in elevated exosome secretion in MDBK (Madin-Darby bovine kidney) cells and an altered microRNA expression profile within the exosomes. Additionally, a substantial enrichment of bta-miR-2484 was identified in the exosome fraction during the course of BoHV-1 infection. Furthermore, it was determined that bta-miR-2484 is substantially enriched in exosomes during BoHV-1 infection, where it promotes the expression of interferon-stimulated genes by activating the JAK-STAT pathway through targeted inhibition of SOCS5. This study proposes a novel perspective on the antiviral activity of bta-miR-2484 in exosomes, suggesting that this miRNA may play a crucial role in the host response to BoHV-1 infection.

## Materials and methods

### Cells and virus

BoHV-1/BarthaNu/67 was obtained from the China Veterinary Medicine Collection Center. The viral titer was determined using the Reed–Muench method and calculated as 10^6.375^TCID_50_ (50% tissue culture infectious dose)/0.1 mL. The MDBK cell lines were obtained from the American Type Culture Collection (ATCC CCL-22) and maintained in our laboratory. The cells were cultivated in 1640 medium (Biosharp, Beijing, China), which was enriched with 10% fetal bovine serum (FBS) (Vazyme, Nanjing, China) at a temperature of 37 °C and 5% CO_2_.

### Isolation and identification of exosomes

MDBK cells were infected with BoHV-1 at a multiplicity of infection (MOI) of 0.1 when they reached approximately 80% confluence. This MOI was calculated based on virus titers determined by the TCID_50_ assay. After 1 h of infection, the culture medium was replaced with fresh medium supplemented with 1% exosome-depleted FBS, and the cells were further cultured for 24 h. Culture supernatants from the control group (C group) and BoHV-1-infected group (B group) were then collected for subsequent experiments. Preliminary processing of cell supernatants was conducted using a differential centrifugation procedure. Briefly, cell culture supernatants were sequentially centrifuged at 4 °C for stepwise impurity removal: 300 *g* for 5 min to remove viable cells, 2000 *g* for 20 min to eliminate dead cells, and 10,000 *g* for 30 min to clear cell debris. The supernatant was then filtered through a 0.22 μm membrane. The filtrate was ultracentrifuged at 100,000 *g* for 2 h, and the supernatant was discarded. The resulting pellet was resuspended in pre-cooled sterile phosphate-buffered saline (PBS) (Biosharp, Beijing, China) and re-ultracentrifuged at 100,000 *g* for 2 h. The purified pellet was finally resuspended in 50 μL of sterile PBS. The harvested exosomes were further purified using a CD63 antibody-labeled exosome capture and isolation kit (Duolaimi, Wuhan, China) in accordance with the manufacturer’s instructions [41].

### Transmission electron microscopy (TEM) and nanoparticle tracking analysis (NTA)

Following the incubation of the prepared exosomes on a 200-mesh copper grid for 10 min, they were stained with a 2% phosphotungstic acid solution for a further 5 min. The samples were allowed to air-dry under incandescent light prior to observing the morphological features of the exosomes using a transmission electron microscope at 80 kV. After appropriately diluting the sample with sterile PBS, particle size and concentration were measured using the ZetaView_Particle Metrix (Particle Metrix, PMX-120, Germany), followed by analysis with NTA software. Prior to utilization, the instrument underwent calibration using 100 nm PS beads [42].

### Cell viability assay

The cytotoxic effect of GW4869 (TargetMol, Boston, MA, USA) on MDBK cells was assessed using the Cell Counting Kit-8 (CCK-8) (Biosharp, Beijing, China). Cells were seeded onto a 96-well cell plate and cultured until they grew into a single layer. The culture medium was removed and cells were washed twice with PBS. The medium was replaced with cell maintenance solution in the presence of dilutions of GW4869. The plates were incubated for 24 h, when the medium was replaced with fresh 1640 containing 10% CCK-8, and the cells were cultured for 1 h, after which absorbance was measured at 450 nm. All assays were performed in triplicate.

### Exosome secretion inhibition assay

MDBK cells were incubated with serially diluted concentrations of GW4869 or vehicle control. Following 24 h of treatment, exosomes were isolated and purified from the respective cell culture supernatants as described above.

### Exosomal small RNA sequencing and target gene prediction

Following the extraction of exosome samples from Group C and Group B using the previously outlined methodology, the desired miRNA was extracted using the miScript miRNA Extraction Kit from the exosome samples. Deep sequencing of three independent sets of exosomal miRNAs per group was performed using the Illumina HiSeq 2500 platform to determine their expression profiles. The prediction of target molecules for differentially expressed microRNAs was conducted using TargetScan [43] and miRanda software [44, 45].

### miRNA, siRNA, and plasmids

 The miRNA-mimics, miRNA-inhibitors, small interfering RNA (siRNA), and plasmids used in this study were synthesized by Suzhou GenePharma Co., Ltd. (Suzhou Gema), with their sequences listed in Additional file 1.

### Dual-luciferase assay

MDBK cells were co-transfected with the SOCS5 mRNA 3′ UTR mutant (Mut) or wild-type (WT) plasmids and the bta-miR-2484 mimics or the miR-NC using Lipofectamine 2000 (Vazyme). Following a 48-h period, the Dual Luciferase Reporter Gene Assay Kit (Beyotime, Shanghai, China) was employed in accordance with the manufacturer’s instructions to detect firefly luciferase and *Renilla* luciferase activity. Firefly luciferase activity was normalized against *Renilla* luciferase activity, which served as an internal control.

### Cell co-culture

The cell co-culture experiments were performed in accordance with the method described by He et al. [46]. Following a 24-h period of transfection, the culture medium was replaced, and the cells were co-cultured with MDBK cells that had been seeded in a six-well plate. Following a 24-h period, the fluorescence signals from the MDBK cells in the six-well plates were observed using a fluorescence microscope.

### The effect of different treatments on BoHV-1 replication

To determine the effects of the SOCS5 protein and bta-miR-2484 on BoHV-1 replication, bta-miR-2484 mimics, mimics-NC, bta-miR-2484 inhibitor, inhibitor-NC, si-SOCS5, si-NC, pcDNA3.1-SOCS5, and pcDNA3.1-NC were transfected into MDBK cells using Lipofectamine 2000. At 24 h post-transfection, cells were infected with BoHV-1 at an MOI of 0.1 and cultured for an additional 24 h. Samples were collected to determine viral titers as described above, while viral copy numbers were quantified using quantitative polymerase chain reaction (qPCR).

### Immunofluorescence

MDBK cells infected with 0.1 MOI of BoHV-1 for 24 h were fixed with 4% paraformaldehyde for 15 min. After permeabilization of the cells with 0.3% Triton X-100 at room temperature for 20 min, they were blocked with 5% bovine serum albumin (BSA) for 1 h. The sample cells were incubated with anti-BoHV-1 glycoprotein E (gE) antibody at a dilution of 1:100 for 2 h at room temperature and then stained with fluorescein isothiocyanate (FITC)-labeled goat anti-rabbit immunoglobulin G (IgG; Abclonal, AS011) for 1 h at room temperature. Finally, the cell nuclei were stained with DAPI at room temperature for 10 min, followed by observation under a fluorescence microscope [47].

### Gene expression analysis

Total RNA was extracted using TRIzol reagent, and complementary DNA (cDNA) was generated through reverse transcription with HiScript III Reverse Transcriptase (Vazyme). Quantitative PCR was conducted using an AceQ Universal SYBR qPCR Master Mix (Vazyme), and the resulting data were analyzed on a LightCycler 96 apparatus (Roche, Basel, Switzerland). To analyze miRNA expression, after extracting miRNAs from exosomes or cells using a miRNA extraction kit (Vazyme), reverse transcription was performed using a miRNA stem-loop reverse transcriptase (Vazyme) and specific stem-loop primers. During the process of isolating exosomal miRNAs, a total of 250 fmol of the synthetic nematode miRNA cel-miR-39 was added to each sample. Finally, qPCR analysis was performed using exogenous cel-miR-39 and endogenous U6 small nuclear RNA as internal controls [44]. The primer sequences used are listed in Additional file 2.

### Western blot

After the exosomes were purified, they were lysed using a lysis buffer specifically designed for exosomal proteins. The supernatant was collected after centrifugation, and the exosomal proteins were obtained by denaturing the sample in 5× loading buffer at 100 °C for 10 min. For cell samples, the collected and processed cells were lysed on ice using radioimmunoprecipitation assay (RIPA) buffer (Beyotime, Shanghai, China) containing 1% phenylmethanesulfonyl fluoride (PMSF) (Beyotime, Shanghai, China) and phosphatase inhibitors. After centrifugation, the supernatant was collected, and the total protein concentration was determined using the BCA (bicinchoninic acid) method and normalized. The cellular proteins were then denatured for 10 min at 100 °C in 5× loading buffer. After separation of equal amounts of protein by 10% sodium dodecyl sulfate–polyacrylamide gel electrophoresis (SDS-PAGE), the proteins were transferred to a 0.2 μm polyvinylidene fluoride (PVDF) membrane. The membrane was blocked with 5% BSA for 1 h and then incubated overnight at 4 °C with the primary antibody: CD63 (Abclonal, A19023), TSG101 (Abclonal, A5789), Alix (Abclonal, A25326), BoHV-1 gE, calnexin (Abclonal, A15631), SOCS5 (Abclonal, A7952), STAT1 (Abclonal, A19563), p-STAT1 (Abclonal, AP0054), β-actin (Abclonal, AC026). The BoHV-1 gE antibody used in this study was generated in our laboratory. Briefly, the recombinant gE protein was expressed using a prokaryotic expression system in *Escherichia coli* BL21 and purified. The purified gE protein was then used to immunize rabbits to generate the polyclonal antibody. After washing with TBST and incubating with horseradish peroxidase (HRP)-conjugated goat anti-rabbit IgG (Abclonal, AS014), protein expression was detected using an enhanced chemiluminescence (ECL) detection system. To prevent signal degradation of the phosphorylated protein caused by the stripping and reprobing process, equal amounts of protein lysates were loaded onto parallel gels to detect total STAT1 and p-STAT1 individually. The expression level of each target protein was subsequently normalized against its corresponding β-actin.

### Statistical analysis

All data in this study were analyzed and plotted using GraphPad Prism 8.0 software and are presented as mean ± standard deviation (SD). For comparisons among multiple groups, a standard one-way analysis of variance (ANOVA) was used; for comparisons between two groups, a one-sample *t*-test was used to analyze the data. A *p*-value less than 0.05 is considered statistically significant.

## Results

### BoHV-1 infection enhances exosome release from MDBK cells

**Figure 1:**
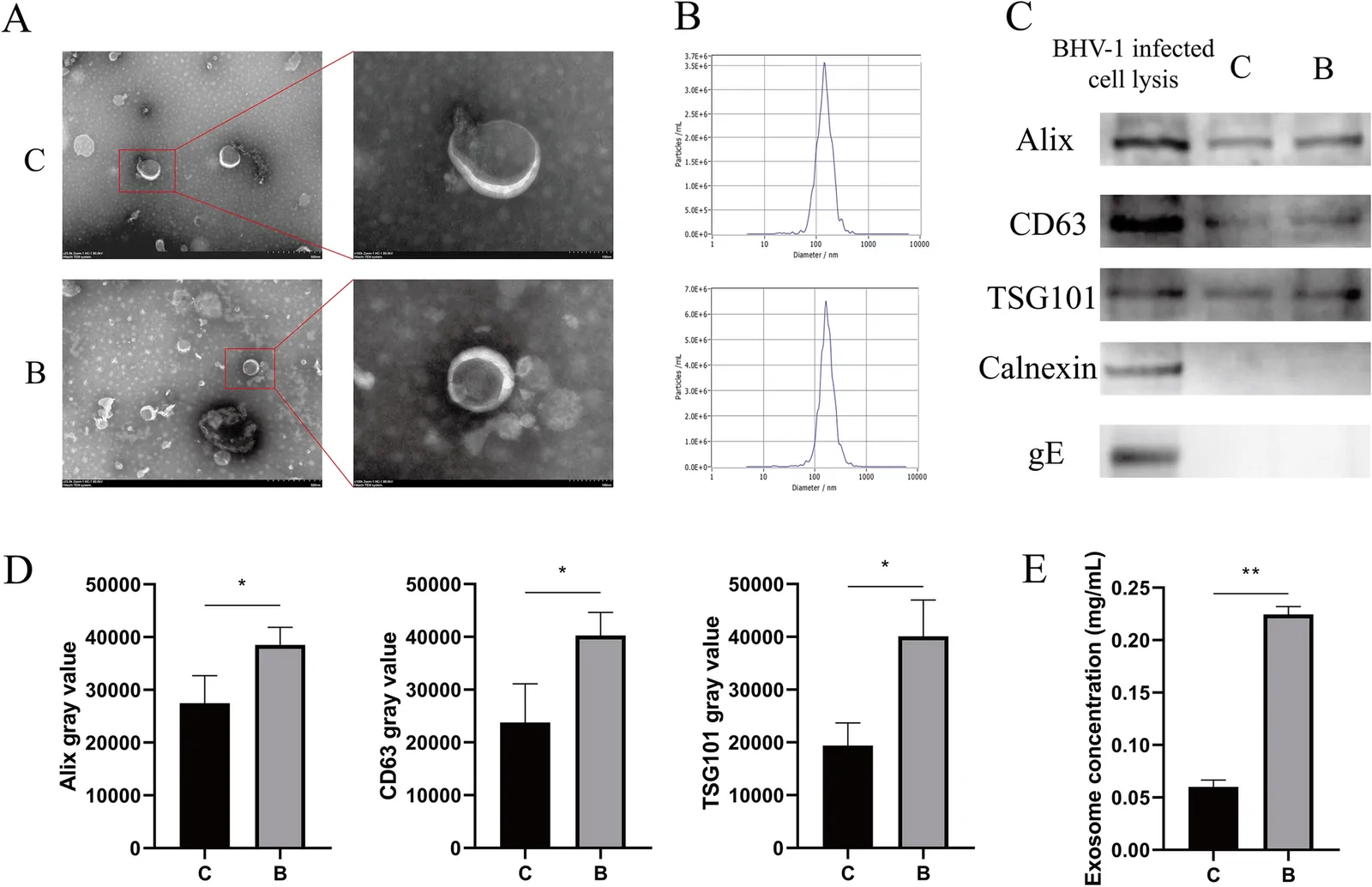
**BoHV-1 infection promotes exosome secretion in MDBK cells**. **A** Transmission electron microscopy (TEM) images of exosomes isolated from the cell culture supernatants of control (Group C) and BoHV-1-infected (Group B) MDBK cells. **B** Nanoparticle tracking analysis (NTA) showing the size distribution of the isolated exosomes. **C** Western blot analysis of exosome marker proteins (Alix, CD63, and TSG101), the endoplasmic reticulum marker calnexin, and the viral protein BoHV-1 gE in infected cell lysates and isolated exosomes. **D** Quantification of Alix, CD63, and TSG101 expression levels based on western blot gray values. **E** Total protein concentration of the isolated exosomes determined via BCA assay. Exosomes were isolated from cell culture supernatants at 24 h post-infection. Data are presented as mean ± SD. *p < 0.05, **p < 0.01.

Exosomes from the cell culture supernatants of different groups were isolated using ultracentrifugation and subsequently purified by immunomagnetic bead-based separation. The TEM study revealed the characteristic exosome structure, characterized by a cup-shaped lipid bilayer vesicle morphology with protrusions at the periphery and a central depression (Figure 1A). It was observed that the diameters of the isolated exosomes ranged from 30 to 200 nm (Figure 1B). The purified exosome samples were characterized by western blotting to confirm their identity. The results demonstrated that the exosome samples from both Groups C and B exhibited the presence of the exosomal marker proteins Alix, CD63, and TSG101 while being negative for the endoplasmic reticulum marker protein calnexin and BoHV-1 gE (Figure 1C). This finding confirms the absence of contamination by cellular structures or viral particles. Furthermore, we inoculated MDBK cells with exosomes derived from each group and cultured them for 72 h. The results showed that none of the exosome treatments induced apparent cytopathic effects (CPE) in MDBK cells. Additionally, qPCR analysis revealed no BoHV-1 replication, which further confirms the high purity of the isolated exosomes (Additional file 3). In order to investigate the effect of BoHV-1 on exosome release, the expression levels of the exosome marker proteins (Alix, CD63, and TSG101) in exosome samples were detected and quantified. The exosome samples were isolated from equal volumes of cell culture supernatants from the two groups, and western blotting was used to detect the expression levels of the marker proteins. The findings of the study indicated that cells infected with BoHV-1 exhibited elevated levels of exosome release (Figure 1D). The protein concentrations of the two groups of isolated exosomes were measured using the BCA protein concentration assay kit. The results obtained were consistent with those from western blotting (Figure 1E). The results of the present study demonstrate that BoHV-1 is able to induce the release of exosomes from MDBK cells.

### The progression of BoHV-1 infection is impeded by the suppression of exosome release

**Figure 2:**
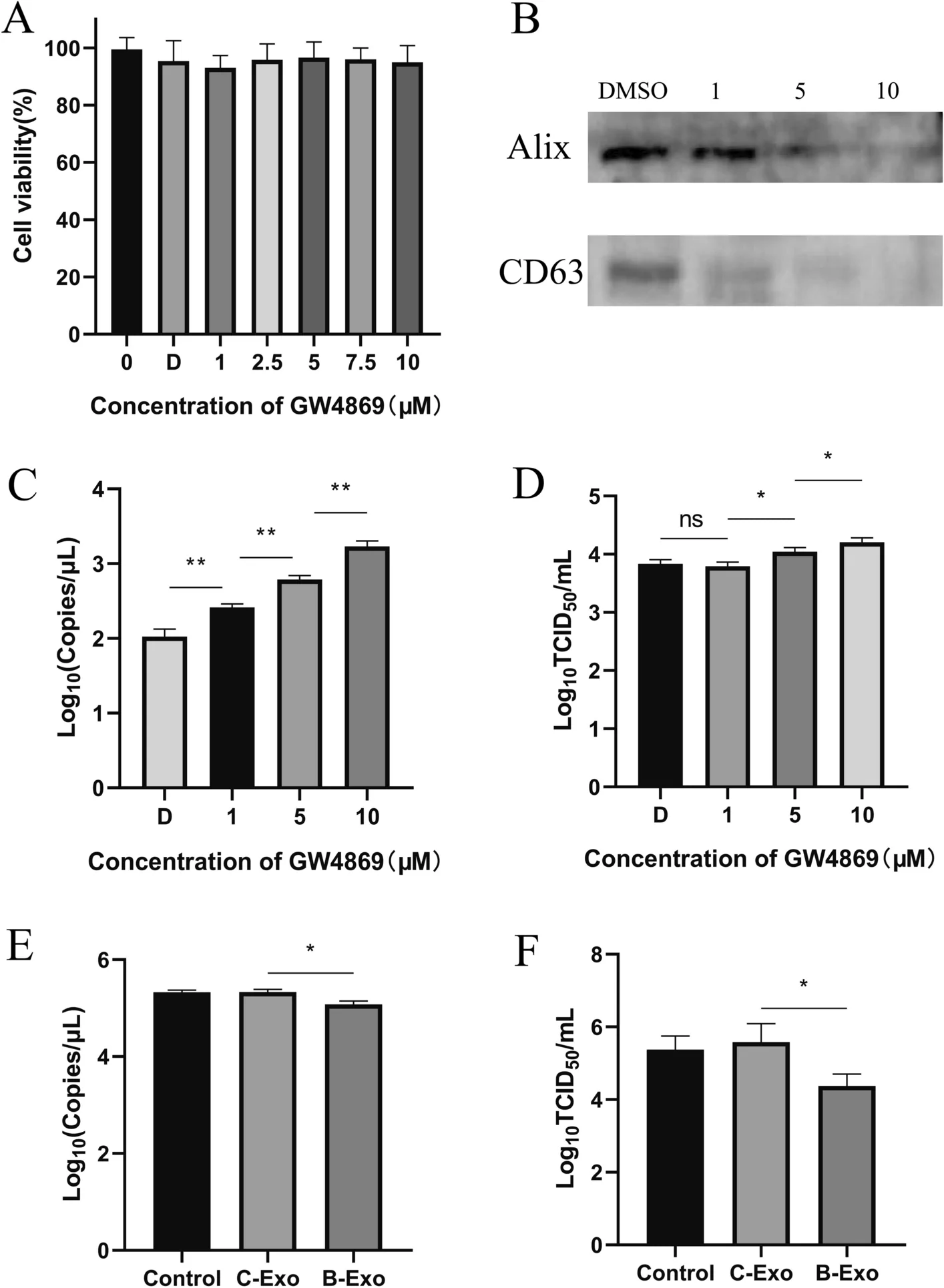
**Inhibition of exosome release promotes BoHV-1 replication**. **A** Cell viability of MDBK cells treated with various concentrations of the exosome inhibitor GW4869 (0, 1, 2.5, 5, 7.5, 10 μM) for 24 h, assessed by CCK-8 assay. **B** Western blot analysis of exosomal markers Alix and CD63 following treatment with varying concentrations of GW4869. **C** BoHV-1 gE copy numbers and **D** viral titers in MDBK cells following treatment with GW4869 (D represents the DMSO control). **E** BoHV-1 gE copy numbers and **F** viral titers in MDBK cells following treatment with exosomes from different groups. For viral infection assays, cells were analyzed at 24 h post-infection. Data are presented as mean ± SD. *p < 0.05, **p < 0.01.

The above results demonstrate that MDBK cells respond to BoHV-1 infection by increasing exosome release. In order to further investigate the role of exosomes in BoHV-1 infection, GW4869, a widely reported inhibitor of exosome biogenesis and release, was utilized to inhibit exosome release from MDBK cells. The assessment of the cytotoxic potential of GW4869 on MDBK cells was conducted utilizing the CCK-8 assay. The findings demonstrated that GW4869 exhibited non-cytotoxic properties even at concentrations as high as 10 μM (Figure 2A). Meanwhile, the levels of the exosomal markers Alix and CD63 were examined by western blotting to evaluate the effect of GW4869 on exosome release. The results indicated that GW4869 suppressed exosome release in a dose-dependent manner (Figure 2B). BoHV-1 replication in MDBK cells was then assessed after treatment with varying concentrations of GW4869. The results demonstrated that as the inhibition of exosomes increased, the proliferation rate of BoHV-1 increased significantly (Figure 2C, D). Subsequently, we treated BoHV-1-infected MDBK cells with exosomes (50 μg/mL) derived from groups C and B, respectively, and evaluated viral replication at 24 h post-treatment (Figure 2E, F). The results demonstrated that exosomes from group B effectively inhibited BoHV-1 replication. The findings indicate that the release of exosomes plays a pivotal role in the suppression of BoHV-1 replication during the course of infection.

### BoHV-1 infection reshapes the exosomal miRNA expression profile

**Figure 3:**
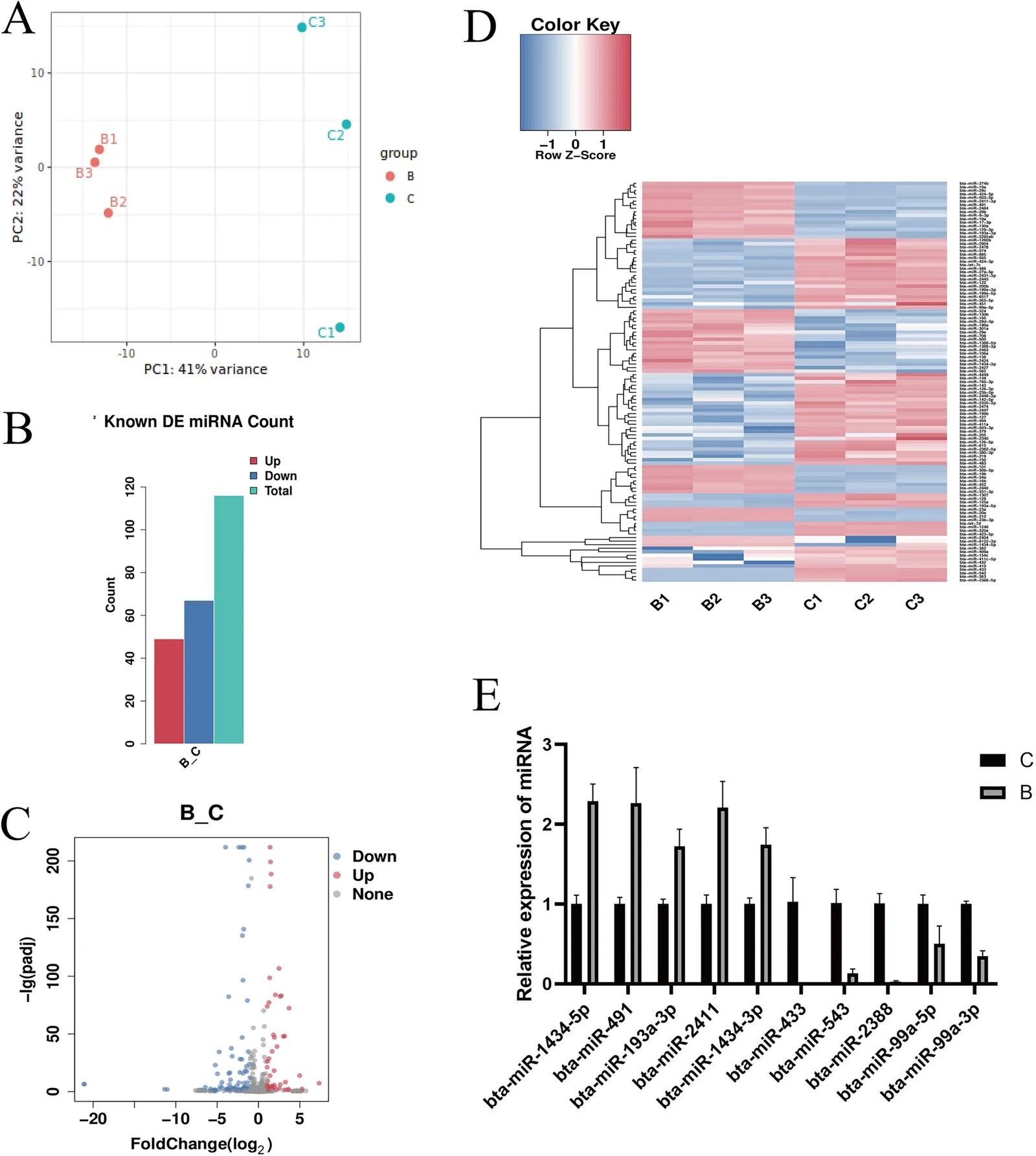
**BoHV-1 infection alters the expression profile of exosomal miRNAs**. **A** Principal component analysis (PCA) of exosomal miRNA sequencing data from Group C and Group B. **B** Bar chart displaying the total count of known differentially expressed (DE) miRNAs. **C** Volcano plot illustrating the distribution and magnitude of differentially expressed miRNAs. **D** Hierarchical clustering heatmap of all differentially expressed miRNAs in exosomes from Group B versus Group C. **E** Validation of selected differentially expressed miRNAs using quantitative PCR. Exosomes were isolated from cell culture supernatants at 24 h post-infection. Data are presented as mean ± SD.

Exosomes have the capacity to transport bioactive miRNAs between cells, thereby inducing alterations or reprogramming of the biological activity of target cells. Studies have shown that miRNAs within exosome complexes are involved in the host’s defense against viral infection. A comprehensive analysis of the expression patterns of miRNAs in two groups of exosomes was undertaken using deep sequencing. The results of the principal component analysis showed that samples from Group B and Group C exhibited distinct separation between groups and clustering within groups, suggesting significant differences in the overall miRNA expression patterns between the two groups (Figure 3A). Furthermore, a total of 116 known microRNAs were found to be differentially expressed in the exosome samples of Group B in comparison with Group C (Figure 3B) (Additional file 4). A volcano plot visualizing the distribution of these differentially expressed miRNAs is shown in Figure 3C. Among 49 upregulated miRNAs, seven were overexpressed with log_2_fold change (log_2_FC) ≥ 3; of 67 downregulated miRNAs, 25 were underexpressed, with log_2_FC ≤ −3. The volcano plot illustrates the magnitude of changes in both miRNA sets. Hierarchical clustering analysis of the differentially expressed miRNAs revealed distinct expression patterns between Group B and Group C, with samples within each group clustering closely together (Figure 3D). Furthermore, the expression levels of selected differentially expressed miRNAs were validated by real-time qPCR, and the results were consistent with the sequencing data (Figure 3E). The results presented herein demonstrate that BoHV-1 infection leads to changes in the expression profile of miRNAs in exosomes.

### BoHV-1 infection induces preferential exosomal sorting of bta-miR-2484 and its transfer to recipient cells

**Figure 4:**
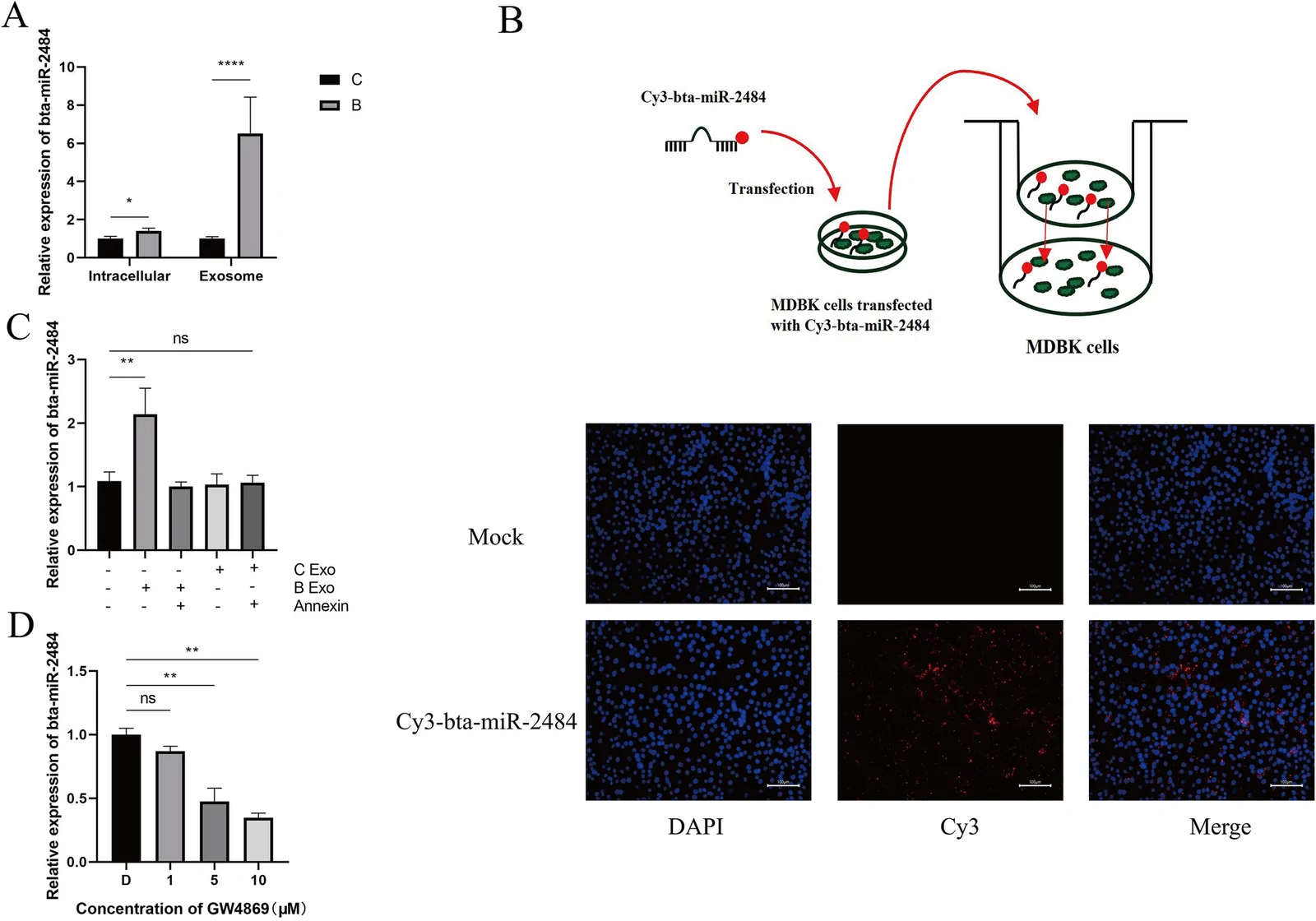
**Preferential sorting of bta-miR-2484 into exosomes and intercellular transfer during BoHV-1 infection**. **A** Relative expression levels of bta-miR-2484 in intracellular and exosomal fractions from control and BoHV-1-infected cells, measured by qPCR. **B** Representative fluorescence images showing the intercellular transfer of Cy3-labeled bta-miR-2484 (red) in a transwell co-culture system. Nuclei were stained with DAPI (blue). **C** Relative bta-miR-2484 expression in recipient cells treated with exosomes from Group C or Group B, with or without annexin V (an exosome internalization inhibitor). **D** Relative bta-miR-2484 expression in recipient cells treated with different concentrations of the exosome release inhibitor GW4869. Data are presented as mean ± SD. **p < 0.01, ****p < 0.0001, ns = nonsignificant.

Since bta-miR-2484 was significantly upregulated in the samples (log_2_ FC > 3 and *p* < 0.01), subsequent studies focused on bta-miR-2484. To ascertain its biological function during BoHV-1 infection, its expression levels were validated using qPCR in conjunction with sequencing results. It was found that bta-miR-2484 was significantly enriched in cells during BoHV-1 infection; moreover, the enrichment of bta-miR-2484 in exosomes was significantly higher than that in cells (Figure 4A). In order to ascertain whether bta-miR-2484 can be transferred between cells via paracrine mechanisms, a co-culture system was employed. MDBK cells in the upper chamber of a co-culture chamber were transfected with Cy3-labeled bta-miR-2484, and then co-cultured with untreated MDBK cells in the lower chamber. Red fluorescence was observed in the cells in the lower chamber (Figure 4B), demonstrating that bta-miR-2484 can be secreted into the extracellular space and taken up by other cells. In the subsequent stage of the experiment, the exosome samples isolated from groups C and B were added to MDBK cells that had been treated with annexin V, a substance that has been shown to inhibit exosome internalization. The results indicated a significant increase in bta-miR-2484 levels in cells treated with exosome preparations from Group B, while this phenomenon remained absent in the C groups (Figure 4C). Furthermore, GW4869 was utilized to inhibit exosome release, and it was observed that as exosome release was suppressed, the levels of bta-miR-2484 in the cells gradually decreased (Figure 4D). While the results for the lower concentration of GW4869 demonstrated no statistically significant difference when compared with the control group, a downward trend was observed, which may be associated with the degree of exosome inhibition. These results show that BoHV-1 infection causes bta-miR-2484 to be sorted into exosomes, which then transport it to other cells.

### bta-miR-2484 restricts BoHV-1 replication in MDBK cells

**Figure 5:**
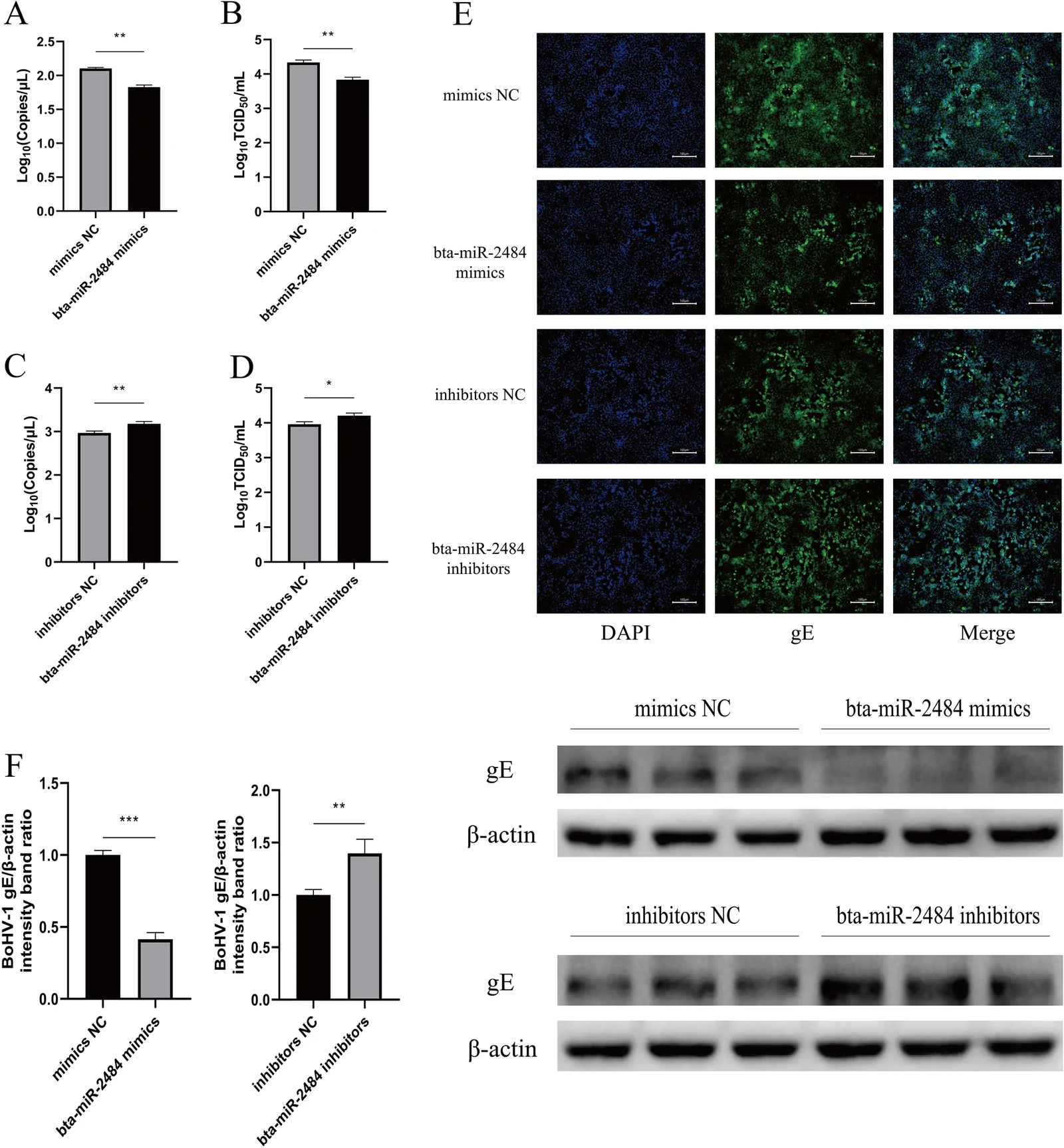
**bta-miR-2484 inhibits BoHV-1 replication in host cells**. **A**, **B** gE copy numbers and viral titers in MDBK cells transfected with bta-miR-2484 mimics or mimic NC followed by BoHV-1 infection. **C**, **D** Viral titers and gE copy numbers in cells transfected with bta-miR-2484 inhibitors or inhibitors NC. **E** Representative immunofluorescence images of BoHV-1 gE expression (green) in MDBK cells transfected with bta-miR-2484 mimics or inhibitors. **F** Western blot analysis of BoHV-1 gE protein expression in MDBK cells transfected with bta-miR-2484 mimics or inhibitors. Data are presented as mean ± SD. *p < 0.05, **p < 0.01, *** *p* < 0.001.

In the aforementioned study, it was confirmed that bta-miR-2484 is transferred to other cells via exosomes during BoHV-1 infection, thereby regulating its levels within those cells. Subsequently, we conducted further investigation into the specific function of bta-miR-2484 in the context of BoHV-1 infection. The MDBK cells were subjected to transfection with bta-miR-2484 mimics or mimics NC (negative control), followed by infection with BoHV-1 at an MOI of 0.1 for a duration of 24 h. The replication of BoHV-1 was then analyzed in both groups by measuring viral titers and copy numbers. The results demonstrated that, in comparison with the mimics NC group, the copy numbers of the BoHV-1 gE gene were markedly diminished in the group transiently expressing bta-miR-2484 mimics (Figure 5A), and the viral titers exhibited a similar tendency (Figure 5B). In order to provide further confirmation of its function, bta-miR-2484 inhibitors were used to suppress the expression of endogenous bta-miR-2484, after which the cells were inoculated with BoHV-1. The results for viral titer and gE gene copy number demonstrated that suppression of bta-miR-2484 significantly increased BoHV-1 replication in MDBK cells (Figure 5C, D). In order to provide further validation of these results, an immunofluorescence assay was performed using BoHV-1 gE antibodies to detect the expression levels of the gE protein. The results demonstrated that the expression of bta-miR-2484 impaired the expression of BoHV-1 gE, thereby inhibiting BoHV-1 replication, whereas the silencing of bta-miR-2484 promoted BoHV-1 replication (Figure 5E). These phenotypic observations were further supported by western blot analysis, which demonstrated consistent alterations in BoHV-1 gE protein expression (Figure 5F). It is evident from these results that bta-miR-2484 is capable of impeding the replication of BoHV-1.

### bta-miR-2484 directly targets SOCS5

**Figure 6:**
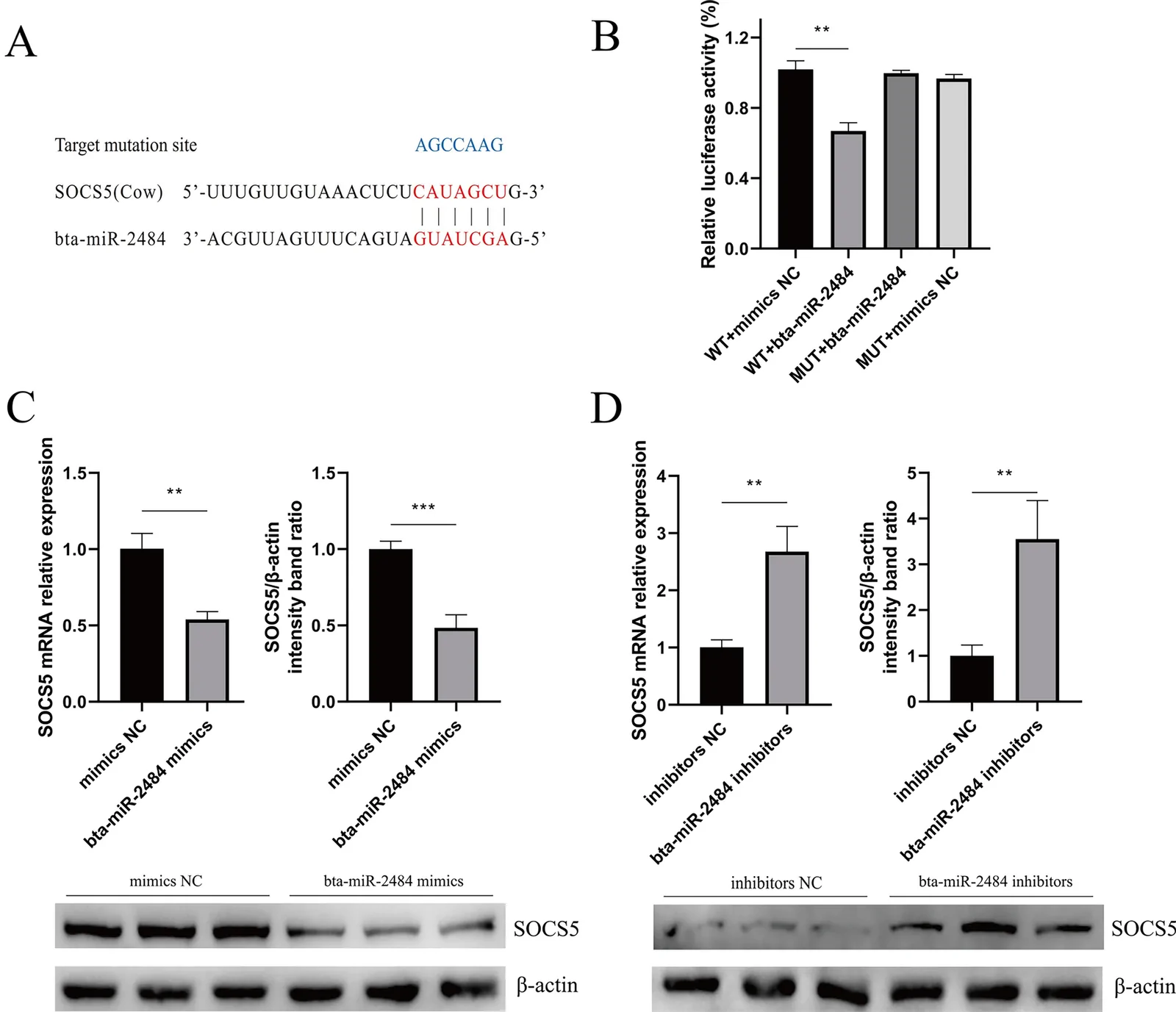
**bta-miR-2484 directly targets the 3′ UTR of SOCS5**. **A** Schematic representation of the predicted binding sites of bta-miR-2484 within the 3′ UTR of bovine SOCS5 mRNA and the designed seed region mutation. **B** Relative luciferase activity in MDBK cells co-transfected with SOCS5 3′ UTR WT or Mut reporter plasmids and bta-miR-2484 mimics or mimics NC. **C** Relative mRNA (qPCR) and protein (western blot) expression levels of SOCS5 in MDBK cells following transfection with bta-miR-2484 mimics. **D** Relative mRNA and protein expression levels of SOCS5 following transfection with bta-miR-2484 inhibitors. Data are presented as mean ± SD. **p < 0.01.

The biological functions of miRNAs are realized through the targeting and regulation of the expression of their specific target genes. The TargetScan and miRanda databases were utilized to identify a putative target site for bta-miR-2484 in the 3′ UTR of SOCS5. In order to verify that SOCS5 is a target of bta-miR-2484 in host cells, a dual-luciferase reporter gene was constructed containing the predicted target site in the 3′ UTR: SOCS5 3′ UTR WT and the target site mutant vector SOCS5 3′ UTR Mut (Figure 6A). In the subsequent experiment, MDBK cells were co-transfected with bta-miR-2484 mimics and either the SOCS5 3′ UTR WT or the SOCS5 3′ UTR Mut plasmid. Following a 24-h period, the luciferase activity was measured using a dual-luciferase assay. The results demonstrated that luciferase activity was significantly reduced in MDBK cells co-transfected with bta-miR-2484 mimics and WT, while activity remained unchanged in the other groups (Figure 6B). Furthermore, following transfection with the mimic, the expression levels of both the SOCS5 mRNA and protein were significantly reduced in comparison with the NC group (Figure 6C), whereas expression levels increased following transfection with the inhibitors (Figure 6D). These results demonstrate that bta-miR-2484 targets the 3′ UTR of SOCS5 to inhibit SOCS5 expression.

### SOCS5 acts as a proviral regulator during BoHV-1 infection

**Figure 7:**
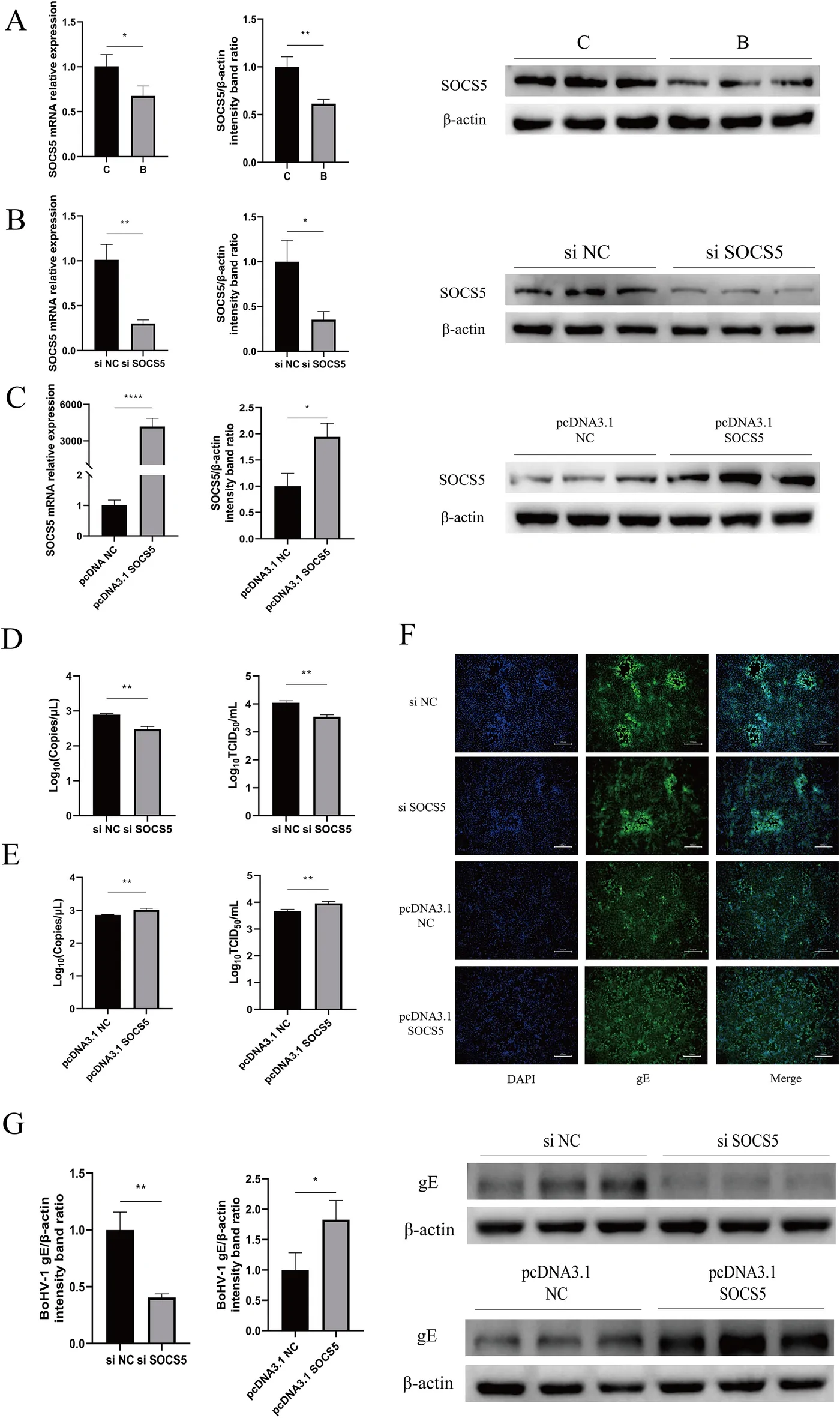
**SOCS5 promotes BoHV-1 replication in MDBK cells**. **A** Relative mRNA and protein expression of SOCS5 in C and B group MDBK cells. **B** Validation of SOCS5 knockdown efficiency using si-SOCS5 via qPCR and western blot. **C** Validation of SOCS5 overexpression efficiency using the pcDNA3.1-SOCS5 plasmid. **D** Viral gE copy numbers and viral titers in BoHV-1-infected cells following SOCS5 knockdown. **E** Viral gE copy numbers and viral titers following SOCS5 overexpression. **F** Representative immunofluorescence images of BoHV-1 gE expression under conditions of SOCS5 knockdown or overexpression. **G** Western blot analysis of BoHV-1 gE protein expression in MDBK cells transfected with siSOCS5 or pcDNA3.1 SOCS5. Data are presented as mean ± SD. *p < 0.05, **p < 0.01, ****p < 0.0001.

To further determine whether SOCS5 participates in the antiviral effect associated with bta-miR-2484 during BoHV-1 infection, we first examined the expression of SOCS5 in BoHV-1-infected MDBK cells. qPCR and western blot analyses showed that BoHV-1 infection significantly reduced SOCS5 expression at both the mRNA and protein levels compared with the control group (Figure 7A). This finding suggests an association between BoHV-1-induced bta-miR-2484 enrichment and the downregulation of SOCS5. We next investigated the functional role of SOCS5 in BoHV-1 replication. MDBK cells were transfected with SOCS5-specific siRNA to knock down SOCS5 expression or with a SOCS5-overexpressing plasmid to increase SOCS5 expression. The efficiency of SOCS5 knockdown and overexpression was confirmed by qPCR and western blot analyses (Figure 7B, C). Following SOCS5 modulation, cells were infected with BoHV-1, and viral replication was assessed 24 h later by measuring viral titers and viral genomic copy numbers. SOCS5 knockdown significantly reduced BoHV-1 titers and gE gene copy numbers compared with the siRNA negative-control group (Figure 7D). In contrast, SOCS5 overexpression markedly increased BoHV-1 titers and viral copy numbers compared with the vector-control group (Figure 7E). Consistent with this, indirect immunofluorescence analysis showed weaker BoHV-1 gE staining in SOCS5-silenced cells, whereas SOCS5 overexpression enhanced gE protein expression (Figure 7F). These findings were further quantitatively corroborated by western blot analysis, demonstrating consistent changes in BoHV-1 gE expression (Figure 7G). Together, these results indicate that SOCS5 facilitates BoHV-1 replication in MDBK cells. Therefore, the suppression of SOCS5 by bta-miR-2484 may contribute to the antiviral response against BoHV-1 infection.

### bta-miR-2484 reduces BoHV-1 replication by inhibiting SOCS5 and activating the JAK-STAT pathway

**Figure 8:**
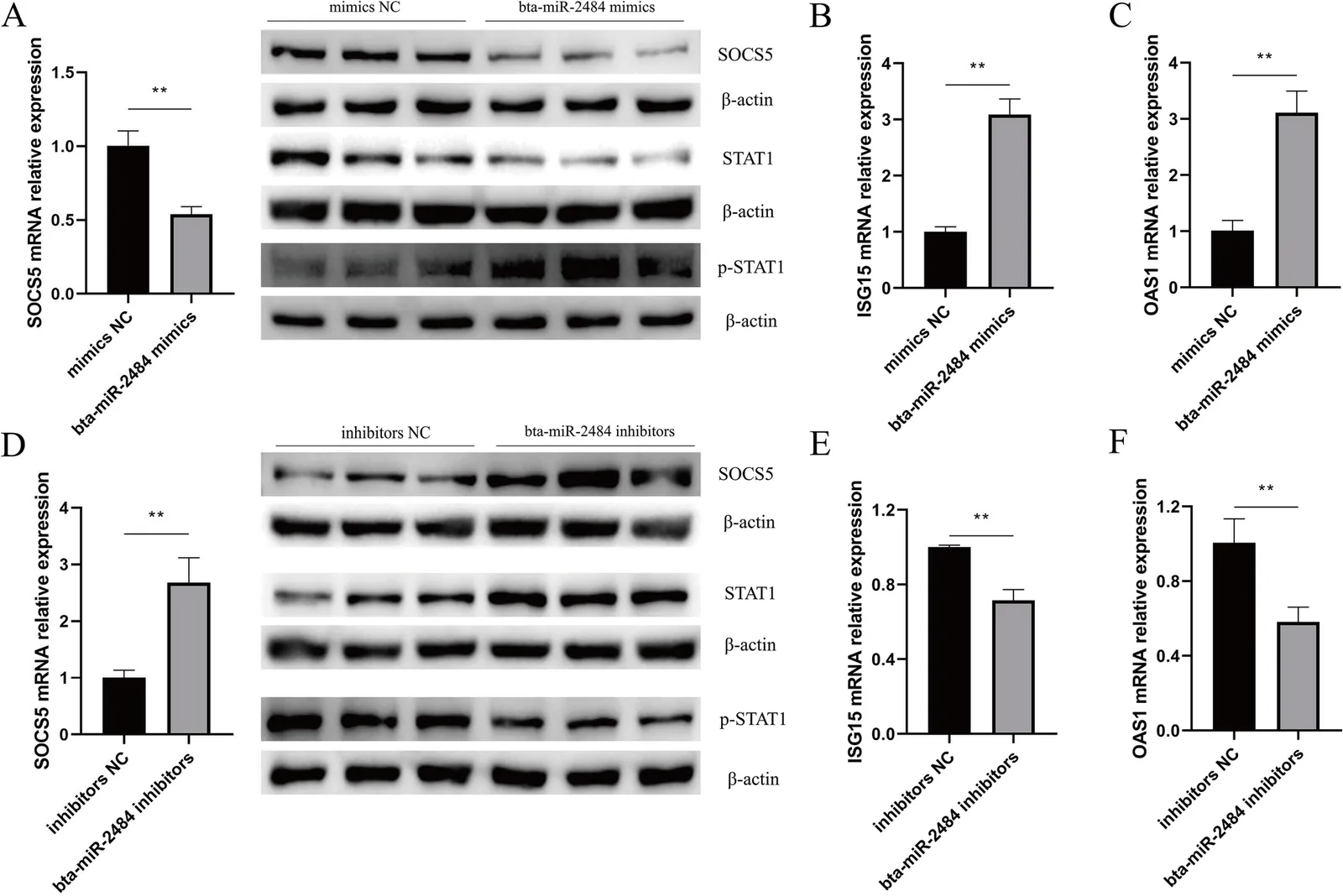
**bta-miR-2484 activates the JAK-STAT pathway and downstream ISGs by downregulating SOCS5**. **A** Western blot analysis of SOCS5, STAT1, and phosphorylated STAT1 (p-STAT1) protein expression in MDBK cells transfected with bta-miR-2484 mimics. **B**, **C** Relative mRNA expression of downstream interferon-stimulated genes ISG15 and OAS1 following transfection with bta-miR-2484 mimics. **D** Western blot analysis of SOCS5, STAT1, and p-STAT1 following transfection with bta-miR-2484 inhibitors. **E**, **F** Relative mRNA expression of ISG15 and OAS1 following transfection with bta-miR-2484 inhibitors. Data are presented as mean ± SD. **p < 0.01.

Given that SOCS5 functions as a negative regulator of the JAK-STAT pathway, we next investigated whether bta-miR-2484 modulates JAK-STAT signaling during BoHV-1 infection. MDBK cells were transfected with bta-miR-2484 mimics or mimic negative control, and the expression of SOCS5 and key components of the JAK-STAT pathway was subsequently examined. Overexpression of bta-miR-2484 markedly reduced SOCS5 expression at both the mRNA and protein levels, while increasing STAT1 phosphorylation without an obvious increase in total STAT1 expression (Figure 8A). Consistent with this, mRNA levels of the interferon-stimulated genes ISG15 and OAS1 were significantly upregulated in cells transfected with bta-miR-2484 mimics compared with the negative-control group (Figure 8B, C). Conversely, inhibition of endogenous bta-miR-2484 produced the opposite effect. Transfection with bta-miR-2484 inhibitors increased SOCS5 expression and reduced STAT1 phosphorylation (Figure 8D). In parallel, the expression levels of ISG15 and OAS1 were significantly decreased in the inhibitor-treated group compared with the inhibitor negative-control group (Figure 8E, F). Together, these results indicate that bta-miR-2484 enhances JAK-STAT pathway activation by suppressing SOCS5 expression, thereby promoting STAT1 phosphorylation and the induction of interferon-stimulated genes. These findings further suggest that bta-miR-2484 restricts BoHV-1 replication, at least in part, by strengthening JAK-STAT-mediated antiviral signaling.

## Discussion

Exosomes are nanosized membrane-bound vesicles actively secreted by cells into the extracellular environment and are increasingly recognized as key mediators of intercellular communication. Their emerging roles in viral replication, host immune modulation, and disease pathogenesis have made them an important focus of virology research [11]. During viral infection, viral particles and exosomes are often released simultaneously into the extracellular milieu, posing a major challenge for studies aimed at defining exosome-specific functions. Thus, reliable separation of exosomes from viral particles is essential for ensuring the accuracy and biological relevance of downstream analyses [48]. The conventional method of ultracentrifugation for exosome isolation has been rendered unsuitable due to its propensity to result in the contamination of viruses. Consequently, alternative methods such as density gradient centrifugation and immunoprecipitation have gained increased prominence in this field [49]. Wang et al. optimized the iodixanol density gradient centrifugation method for the separation of rabies virus and exosomes [50]. Fu et al. successfully isolated exosomes from EV71 using a polyethylene glycol (PEG) precipitation method combined with immunoprecipitation [16]. Given the similarity in buoyant density exhibited by herpesviruses and exosome samples, the application of density gradient centrifugation may no longer be appropriate [51]. Consequently, we opted for an alternative method involving ultracentrifugation in conjunction with immunoprecipitation to purify exosome samples free from any contamination by BoHV-1. Viral infection has been demonstrated to induce alterations in the quantity and properties of exosome secretion by cells relative to their levels in normal cells. These changes facilitate the transfer of antiviral components and help to limit viral spread [13, 52]. In their study of rabies vaccines, Wang et al. also found that rabies virus infection of MRC-5 cells increases the secretion of their exosomes [44]. In the course of research conducted on herpesviruses, it has been determined that cells infected with HSV-1 release STING-containing exosomes that are transmitted between cells, thereby triggering a type I interferon response [53]. It is further postulated that other species within this family may also be involved in the secretion or regulation of extracellular vesicles [11]. The findings of this study demonstrate that BoHV-1 infection induces a substantial increase in exosome secretion, a process which could potentially amplify its own transmission or elicit an immune response initiated by the virus itself. In order to provide further validation of the effect of the substance on BoHV-1 replication, a study was performed in which exosome release was graded and found to be negatively correlated with the extent of BoHV-1 replication. More importantly, exosomes derived from BoHV-1-infected cells exhibited a significant inhibitory effect on viral replication. These results suggest that MDBK cells may trigger antiviral responses in other cells by releasing increased amounts of exosomes that carry certain substances. Due to the complex composition of exosomes, this inhibitory effect may be caused by miRNAs, interferons, viral or cellular proteins, or other bioactive components carried by the exosomes. Specifically regarding viral proteins, while we confirmed the absence of gE to rule out intact viral particle contamination, previous investigators have demonstrated the incorporation of various BoHV-1 proteins into extracellular vesicles derived from infected cells [54]. Therefore, we cannot exclude the possibility that additional viral proteins contribute to the observed inhibitory activity. Nevertheless, considering that miRNAs constitute the most abundant functional nucleic acids in exosomes and serve as critical post-transcriptional regulators of host antiviral immunity, they hold a paramount position as players within this complex cargo. Consequently, miRNAs became the primary focus of our further investigation.

Following secretion into the extracellular space, exosomes facilitate intercellular signaling by transporting their internal contents, which include proteins, mRNA, and miRNA [55]. The composition of these substances is subject to alteration in response to stressors such as bacterial and viral infections and cancer [56]. MiRNAs represent the most abundant type of RNA in exosomes, and as such have attracted significant attention due to their high conservation across species and their extensive regulatory role in gene expression [55]. The expression profile of miRNA in the exosome fraction of chickens infected with the H5N1 avian influenza virus exhibited significant changes; researchers identified a total of 20 miRNAs that exhibited significantly different expression patterns [52]. Truong’s study identified a total of 59 differentially expressed miRNAs in serum exosome samples from cattle infected with lumpy skin disease virus (LSDV) [57]. A deep sequencing analysis of miRNA in exosome samples derived from MDBK cells infected with BoHV-1 was conducted, leading to the identification of 116 significantly differentially expressed miRNAs. This comprehensive analysis provides deep insight into the molecular changes MDBK cells undergo in response to BoHV-1 infection. Previous studies have demonstrated that a number of miRNAs can exert a substantial influence on the replication of BoHV-1. Hou et al. reported that bta-miR-2361 directly targets EGR1 to inhibit BoHV-1 replication [23]. Kanokudom et al. also found that although bhv1-miR-B8-3p is an miRNA encoded by BoHV-1 itself, it exerts a certain inhibitory effect on BoHV-1 replication, which may be related to latent BoHV-1 infection [58]. The role and mechanism of bta-miR-2484, one of the differentially expressed genes identified in our results, in viral infection and replication have not yet been evaluated. In the context of BoHV-1 infection, it was found that bta-miR-2484 is significantly more enriched in the exosomes than in the cells, and can be transferred to recipient cells via exosomes. At the same time, the inhibitory effect of bta-miR-2484 mimics on BoHV-1 replication and the promotional effect of bta-miR-2484 inhibitors fully demonstrate their impact on viral replication.

A mounting body of evidence indicates that cellular miRNAs fulfill their biological functions by modulating the expression of multiple genes through binding to the 3′ UTR of target genes [59]. To ascertain the specific gene to which bta-miR-2484 exerts its inhibitory effect, TargetScan and miRanda prediction tools were utilized to identify genes containing the bta-miR-2484 seed sequence within their 3′ UTR. Combining the results from the dual-luciferase reporter assay and western blot analysis, it was ultimately determined that SOCS5 is a key target gene. BoHV-1 infection has been demonstrated to induce a natural immune response in the host, with the type I interferon signaling pathway serving as a critical line of defense against viral infection [60]. When a virus invades a host, the cells produce large amounts of interferon, which activates the expression of downstream interferon-stimulated genes (ISGs) and inhibits viral replication [61]. The JAK-STAT pathway is a key pathway for transmitting signals of viral infection, and the SOCS family plays a crucial role in the negative feedback regulation of cytokine signaling. Numerous studies have demonstrated that miRNAs influence pathogen infection by regulating the JAK-STAT pathway through the inhibition of the SOCS protein family. Zhang et al. demonstrated that miR-101 promotes the suppression of FHV-1 replication by type I interferons through its targeting of SOCS5 [47]. Wang et al. discovered that gga-miR-365-3p targets SOCS5, thus facilitating *Mycoplasma gallisepticum* evasion of the host’s immune surveillance [62]. To date, only a limited number of studies have been reported on the effects of SOCS5 on BoHV-1. The present study has demonstrated that, in the presence of BoHV-1, bta-miR-2484 accumulates in the exosomes. Following transfer to recipient cells, the target of this microRNA is SOCS5, with the result that its expression is inhibited. This, in turn, promotes STAT1 phosphorylation and activates the JAK-STAT pathway, leading to elevated expression of ISG15 and OAS1. These ISGs are broad-spectrum antiviral effectors; they have been shown to inhibit several alphaherpesviruses and may restrict BoHV-1 replication [47, 63–65].

## Conclusion

In summary, we have successfully demonstrated, for the first time, the regulatory role and mechanisms of both exosomes and bta-miR-2484 in the proliferation of BoHV-1. During BoHV-1 infection, MDBK cells increase exosome release and alter the expression profile of their internal miRNAs, resulting in significant enrichment of bta-miR-2484 in exosomes and its subsequent transfer to recipient cells. Concurrently, bta-miR-2484 was identified as a regulator of SOCS5, a pivotal element within the antiviral signaling pathway. Through the suppression of SOCS5 expression, the JAK-STAT pathway is activated, leading to the enhancement of interferon-stimulated gene expression and the inhibition of BoHV-1 replication. This study provides substantial evidence confirming the important role of exosomes in the cellular defense against BoHV-1 infection. This will enhance our understanding of the molecular mechanisms by which miRNAs in exosomes regulate viral replication, and offers novel insights into host–virus interactions.

## Supplementary Information

**Additional file 1. Sequences of miRNA mimics, inhibitors and siRNAs**.

**Additional file 2. Sequences of primers used in the research**.

**Additional file 3. Evaluation of viral contamination in the purified exosome preparations.**

**Additional file 4. Summary of all differentially expressed miRNAs.**

## Data Availability

No datasets were generated or analyzed during the current study.

## References

[1] Leite F, Kuckleburg C, Atapattu D, Schultz R, Czuprynski CJ (2004) BHV-1 infection and inflammatory cytokines amplify the interaction of *Mannheimia haemolytica* leukotoxin with bovine peripheral blood mononuclear cells in vitro. Vet Immunol Immunopathol 99:193–202 10.1016/j.vetimm.2004.02.004 15135985

[2] Boubaker-Elandalousi R, Mekni-Toujani M, Kaabi B, Larbi I, Diouani MF, Gharbi M, Akkari H, B’chir F, Ghram A (2014) Non-cytotoxic* Thymus capitata* extracts prevent bovine herpesvirus-1 infection in cell cultures. BMC Vet Res 10:231 10.1186/s12917-014-0231-6 PMC4189610 25260761

[3] Chowdhury SI (1995) Molecular basis of antigenic variation between the glycoproteins C of respiratory bovine herpesvirus 1 (BHV-1) and neurovirulent BHV-5. Virology 213:558–568 10.1016/s0042-6822(95)80137-5 7491780

[4] Liu Y, Cui J, Kang J, Wang Z, Xu X, Wu F (2023) Bovine herpesvirus-1 gE protein inhibits IFN-β production to enhance replication by promoting MAVS ubiquitination and interfering with the interaction between IRF3 and CBP/p300. Vet Microbiol 287:109899 10.1016/j.vetmic.2023.109899 37931576

[5] Madin SH, York CJ, Mckercher DG (1956) Isolation of the infectious bovine rhinotracheitis virus. Science 124:721–722 10.1126/science.124.3225.721 13371311

[6] Huck RA, Cartwright SF (1964) Isolation and classification of viruses from cattle during outbreaks of mucosal or respiratory disease and from herds with reproductive disorders. J Comp Pathol Ther 74:346–365 10.1016/s0368-1742(64)80041-2 14198345

[7] Godhardt-Cooper JA, Zoromski J, Toohey-Kurth K (2009) Evaluation of a blocking enzyme-linked immunosorbent assay for serological diagnosis of Bovine herpesvirus 1. J Vet Diagn Invest 21:523–526 10.1177/104063870902100416 19564503

[8] Fu Y, Xiong S (2023) Exosomes mediate coxsackievirus B3 transmission and expand the viral tropism. PLoS Pathog 19:e1011090 10.1371/journal.ppat.1011090 PMC9888687 36634130

[9] He C, Zheng S, Luo Y, Wang B (2018) Exosome theranostics: biology and translational medicine. Theranostics 8:237–255 10.7150/thno.21945 PMC5743472 29290805

[10] Guo R, Xu J, Li C, Mo C, Ou M (2025) Visualizing and analyzing global knowledge maps and emerging research trends in tumor-derived exosomes using CiteSpace. Discov Oncol 16:1623 10.1007/s12672-025-03416-z PMC12378789 40853581

[11] Anderson MR, Kashanchi F, Jacobson S (2016) Exosomes in viral disease. Neurotherapeutics 13:535–546 10.1007/s13311-016-0450-6 PMC4965413 27324390

[12] Liao TL, Chen YM, Hsieh SL, Tang KT, Chen DY, Yang YY, Liu HJ, Yang SS (2021) Hepatitis C virus-induced exosomal microRNAs and Toll-Like receptor 7 polymorphism regulate B-cell activating factor. mBio 12:e0276421 10.1128/mBio.02764-21 PMC8561394 34724826

[13] Qin W, Jiang J, Wu J, Xie Y, Wu Z, Sun M, Bao W (2024) Exosomal ssc-miR-1343 targets FAM131C to regulate porcine epidemic diarrhea virus infection in pigs. Vet Res 55:91 10.1186/s13567-024-01345-3 PMC11264985 39039559

[14] Li J, Liu K, Liu Y, Xu Y, Zhang F, Yang H, Liu J, Pan T, Chen J, Wu M, Zhou X, Yuan Z (2013) Exosomes mediate the cell-to-cell transmission of IFN-α-induced antiviral activity. Nat Immunol 14:793–803 10.1038/ni.2647 23832071

[15] Xu G, Yan H, Zhu Y, Xie Z, Zhang R, Jiang S (2023) Duck hepatitis A virus type 1 transmission by exosomes establishes a productive infection in vivo and in vitro. Vet Microbiol 277:109621 10.1016/j.vetmic.2022.109621 36525908

[16] Fu Y, Zhang L, Zhang F, Tang T, Zhou Q, Feng C, Jin Y, Wu Z (2017) Exosome-mediated miR-146a transfer suppresses type I interferon response and facilitates EV71 infection. PLoS Pathog 13:e1006611 10.1371/journal.ppat.1006611 PMC5614653 28910400

[17] Li X, Xu B, Moran MS, Zhao Y, Su P, Haffty BG, Shao C, Yang Q (2012) 53BP1 functions as a tumor suppressor in breast cancer via the inhibition of NF-κB through miR-146a. Carcinogenesis 33:2593–2600 10.1093/carcin/bgs298 23027628

[18] Ma W, Wang H, He H (2020) Bta-miR-2890 up-regulates JAK-STAT pathway to inhibit BoHV-1 replication by targeting viral gene UL41. Vet Microbiol 245:108709 10.1016/j.vetmic.2020.108709 32456827

[19] Lee RC, Feinbaum RL, Ambros V (1993) The *C. elegans* heterochronic gene lin-4 encodes small RNAs with antisense complementarity to lin-14. Cell 75:843–854 10.1016/0092-8674(93)90529-y 8252621

[20] Kincaid RP, Sullivan CS (2012) Virus-encoded microRNAs: an overview and a look to the future. PLoS Pathog 8:e1003018 10.1371/journal.ppat.1003018 PMC3534370 23308061

[21] Boss IW, Renne R (2010) Viral miRNAs: tools for immune evasion. Curr Opin Microbiol 13:540–545 10.1016/j.mib.2010.05.017 PMC2920354 20580307

[22] Virzì GM, Clementi A, Zanella M, Ronco C (2025) Non-protein-coding RNA and acute kidney injury: new developments from pathogenesis to potential biomarker. Genes 16:1194 10.3390/genes16101194 PMC12564631 41153411

[23] Hou P, Zhao M, He W, He H, Wang H (2019) Cellular microRNA bta-miR-2361 inhibits bovine herpesvirus 1 replication by directly targeting EGR1 gene. Vet Microbiol 233:174–183 10.1016/j.vetmic.2019.05.004 31176405

[24] Lu J, Getz G, Miska EA, Alvarez-Saavedra E, Lamb J, Peck D, Sweet-Cordero A, Ebert BL, Mak RH, Ferrando AA, Downing JR, Jacks T, Horvitz HR, Golub TR (2005) MicroRNA expression profiles classify human cancers. Nature 435:834–838 10.1038/nature03702 15944708

[25] Hirschberger S, Hinske LC, Kreth S (2018) MiRNAs: dynamic regulators of immune cell functions in inflammation and cancer. Cancer Lett 431:11–21 10.1016/j.canlet.2018.05.020 29800684

[26] Hydbring P, Badalian-Very G (2013) Clinical applications of microRNAs. F1000Res 2:136 10.12688/f1000research.2-136.v1 PMC3917658 24627783

[27] Schulte C, Karakas M, Zeller T (2017) microRNAs in cardiovascular disease – clinical application. Clin Chem Lab Med 55:687–704 10.1515/cclm-2016-0576 27914211

[28] Condrat CE, Thompson DC, Barbu MG, Bugnar OL, Boboc A, Cretoiu D, Suciu N, Cretoiu SM, Voinea SC (2020) MiRNAs as biomarkers in disease: latest findings regarding their role in diagnosis and prognosis. Cells 9:276 10.3390/cells9020276 PMC7072450 31979244

[29] Pegtel DM, Gould SJ (2019) Exosomes. Annu Rev Biochem 88:487–514 10.1146/annurev-biochem-013118-111902 31220978

[30] Mashouri L, Yousefi H, Aref AR, Ahadi AM, Molaei F, Alahari SK (2019) Exosomes: composition, biogenesis, and mechanisms in cancer metastasis and drug resistance. Mol Cancer 18:75 10.1186/s12943-019-0991-5 PMC6444571 30940145

[31] Han Z, Liu X, Chen X, Zhou X, Du T, Roizman B, Zhou G (2016) MiR-H28 and miR-H29 expressed late in productive infection are exported and restrict HSV-1 replication and spread in recipient cells. Proc Natl Acad Sci U S A 113:E894-901 10.1073/pnas.1525674113 PMC4763765 26831114

[32] Yang L, Zhang YJ (2017) Antagonizing cytokine-mediated JAK-STAT signaling by porcine reproductive and respiratory syndrome virus. Vet Microbiol 209:57–65 10.1016/j.vetmic.2016.12.036 PMC7117332 28069291

[33] Randall RE, Goodbourn S (2008) Interferons and viruses: an interplay between induction, signalling, antiviral responses and virus countermeasures. J Gen Virol 89:1–47 10.1099/vir.0.83391-0 18089727

[34] Raftery N, Stevenson NJ (2017) Advances in anti-viral immune defence: revealing the importance of the IFN JAK/STAT pathway. Cell Mol Life Sci 74:2525–2535 10.1007/s00018-017-2520-2 PMC7079803 28432378

[35] Huang S, Liu K, Cheng A, Wang M, Cui M, Huang J, Zhu D, Chen S, Liu M, Zhao X, Wu Y, Yang Q, Zhang S, Ou X, Mao S, Gao Q, Yu Y, Tian B, Liu Y, Zhang L, Yin Z, Jing B, Chen X, Jia R (2020) SOCS proteins participate in the regulation of innate immune response caused by viruses. Front Immunol 11:558341 10.3389/fimmu.2020.558341 PMC7544739 33072096

[36] Cui R, Zhang J (2025) Research progress of SOCS in autoimmune diseases: mechanisms and therapeutic implications. Front Immunol 16:1604271 10.3389/fimmu.2025.1604271 PMC12313687 40755762

[37] Pothlichet J, Chignard M, Si-Tahar M (2008) Cutting edge: innate immune response triggered by influenza A virus is negatively regulated by SOCS1 and SOCS3 through a RIG-I/IFNAR1-dependent pathway. J Immunol 180:2034–2038 10.4049/jimmunol.180.4.2034 18250407

[38] Aparecida Silva Barbosa A, Freitas Versiani A, Da Cunha F, Sousa L, Silva De Miranda A, Gasparini MR, Brant F, Silva DG, Luisa Quintino-De-Carvalho I, Marianetti Soriani F, Guimarães Da Fonseca F, César Vasconcelos A, Da Silva BL, Martins Teixeira M, Lúcio Teixeira A, Machado FS, Barbosa-Stancioli EF, Rachid MA (2016) Role of the suppressor of cytokine signaling 2 (SOCS2) during meningoencephalitis caused by Bovine herpesvirus 5 (BoHV-5). Comp Immunol Microbiol Infect Dis 47:26–31 10.1016/j.cimid.2016.05.003 27477504

[39] Sun L, Liu L, Liang D, Liu L (2024) SOCS5, targeted by miR-155-5p, plays a negative regulatory role in pulmonary hypertension through inhibiting JAK2/STAT3 signaling pathway. BMC Pulm Med 24:52 10.1186/s12890-024-02857-6 PMC10809471 38267898

[40] Collison AM, Sokulsky LA, Kepreotes E, Pereira De Siqueira A, Morten M, Edwards MR, Walton RP, Bartlett NW, Yang M, Nguyen TH, Johnston SL, Foster PS, Mattes J (2021) miR-122 promotes virus-induced lung disease by targeting SOCS1. JCI Insight 6:e127933 10.1172/jci.insight.127933 PMC8119205 33830082

[41] Ding T, Cheng T, Zhu X, Xiao W, Xia S, Fang L, Fang P, Xiao S (2023) Exosomes mediate the antibody-resistant intercellular transmission of porcine epidemic diarrhea virus. Vet Microbiol 284:109834 10.1016/j.vetmic.2023.109834 37536161

[42] Jin C, Li K, Yuan H, Zhang X, Rahim MF, Lu Y, Mu S, Wu S, Su H, He X, Yi Z, Yin H, Li J (2026) Outer membrane vesicles derived from yak isolates of *Pasteurella multocida* exhibit promising vaccine potential. Animals (Basel) 16:1264 10.3390/ani16081264 PMC13113515 42072030

[43] TargetScan. http://www.targetscan.org. Accessed 20 Jun 2024

[44] Wang J, Teng Y, Zhao G, Li F, Hou A, Sun B, Kong W, Gao F, Cai L, Jiang C (2019) Exosome-mediated delivery of inducible miR-423-5p enhances resistance of MRC-5 cells to rabies virus infection. Int J Mol Sci 20:1537 10.3390/ijms20071537 PMC6479321 30934732

[45] miRanda. http://www.microna.org/microrna/home.do. Accessed 20 Jun 2024

[46] He L, Zhu W, Chen Q, Yuan Y, Wang Y, Wang J, Wu X (2019) Ovarian cancer cell-secreted exosomal miR-205 promotes metastasis by inducing angiogenesis. Theranostics 9:8206–8220 10.7150/thno.37455 PMC6857047 31754391

[47] Zhang J, Li Z, Huang J, Chen S, Yin H, Tian J, Qu L (2020) miR-101 inhibits feline herpesvirus 1 replication by targeting cellular suppressor of cytokine signaling 5 (SOCS5). Vet Microbiol 245:108707 10.1016/j.vetmic.2020.108707 32456815

[48] Li P, Kaslan M, Lee SH, Yao J, Gao Z (2017) Progress in exosome isolation techniques. Theranostics 7:789–804 10.7150/thno.18133 PMC5327650 28255367

[49] Wang C, Li Z, Liu Y, Yuan L (2021) Exosomes in atherosclerosis: performers, bystanders, biomarkers, and therapeutic targets. Theranostics 11:3996–4010 10.7150/thno.56035 PMC7914371 33664877

[50] Wang J, Wu F, Liu C, Dai W, Teng Y, Su W, Kong W, Gao F, Cai L, Hou A, Jiang C (2019) Exosomes released from rabies virus-infected cells may be involved in the infection process. Virol Sin 34:59–65 10.1007/s12250-019-00087-3 PMC6420551 30725320

[51] Yang D, Zhang W, Zhang H, Zhang F, Chen L, Ma L, Larcher LM, Chen S, Liu N, Zhao Q, Tran PHL, Chen C, Veedu RN, Wang T (2020) Progress, opportunity, and perspective on exosome isolation - efforts for efficient exosome-based theranostics. Theranostics 10:3684–3707 10.7150/thno.41580 PMC7069071 32206116

[52] Hong Y, Truong AD, Lee J, Vu TH, Lee S, Song KD, Lillehoj HS, Hong YH (2021) Exosomal miRNA profiling from H5N1 avian influenza virus-infected chickens. Vet Res 52:36 10.1186/s13567-021-00892-3 PMC7931527 33658079

[53] Dogrammatzis C, Saud R, Waisner H, Lasnier S, Suma SM, Grieshaber B, Kalamvoki M (2024) Tracing the STING exocytosis pathway during herpes viruses infection. mBio 15:e0037324 10.1128/mbio.00373-24 PMC11005388 38470056

[54] Luo Y, Yang H, Huang Y, Goreham RV, Ding X, Zhu L (2025) Extracellular vesicles released by bovine alphaherpesvirus 1-infected A549 cells may limit subsequent infections of the progeny virus. Int J Mol Sci 26:6181 10.3390/ijms26136181 PMC12249866 40649956

[55] Valadi H, Ekström K, Bossios A, Sjöstrand M, Lee JJ, Lötvall JO (2007) Exosome-mediated transfer of mRNAs and microRNAs is a novel mechanism of genetic exchange between cells. Nat Cell Biol 9:654–659 10.1038/ncb1596 17486113

[56] Corrado C, Raimondo S, Chiesi A, Ciccia F, De Leo G, Alessandro R (2013) Exosomes as intercellular signaling organelles involved in health and disease: basic science and clinical applications. Int J Mol Sci 14:5338–5366 10.3390/ijms14035338 PMC3634447 23466882

[57] Truong AD, Tran HTT, Phan L, Phan TH, Chu NT, Vu TH, Nguyen HM, Nguyen LP, Kim C, Dang HV, Hong YH (2025) Differentially expressed miRNA profiles in serum-derived exosomes from cattle infected with lumpy skin disease virus. Pathogens 14:176 10.3390/pathogens14020176 PMC11858326 40005551

[58] Kanokudom S, Mahony TJ, Smith DR, Assavalapsakul W (2018) Modulation of bovine herpesvirus 1 infection by virally encoded microRNAs. Virus Res 257:1–6 10.1016/j.virusres.2018.08.022 30193942

[59] Silva VA, Polesskaya A, Sousa TA, Corrêa VM, André ND, Reis RI, Kettelhut IC, Harel-Bellan A, De Lucca FL (2011) Expression and cellular localization of microRNA-29b and RAX, an activator of the RNA-dependent protein kinase (PKR), in the retina of streptozotocin-induced diabetic rats. Mol Vis 17:2228–2240 PMC3164688 21897745

[60] Afroz S, Brownlie R, Fodje M, Van Drunen Littel-Van Den Hurk S (2016) VP8, the major tegument protein of bovine herpesvirus 1, interacts with cellular STAT1 and inhibits interferon beta signaling. J Virol 90:4889–4904 10.1128/JVI.00017-16 PMC4859699 26889034

[61] Schneider WM, Chevillotte MD, Rice CM (2014) Interferon-stimulated genes: a complex web of host defenses. Annu Rev Immunol 32:513–545 10.1146/annurev-immunol-032713-120231 PMC4313732 24555472

[62] Wang Y, Han Y, Wang L, Zou M, Sun Y, Sun H, Guo Q, Peng X (2022) *Mycoplasma gallisepticum* escapes the host immune response via gga-miR-365-3p/SOCS5/STATs axis. Vet Res 53:103 10.1186/s13567-022-01117-x PMC9721073 36471418

[63] Da Fonseca RN, De Andrade Lourenço D, Zandoná GP, Bortolatto CF, Brüning CA, Kremer FS, Merchioratto I, Maggioli MF, Gressler RP, Freitag RA, Lüdtke R, Hall TK, Bauermann FV, De Oliveira Hübner S (2026) Pampa biome plant extracts and flavonoids show antiviral activity against bovine herpesvirus and bovine viral diarrhea virus. Sci Rep 16:21892 10.1038/s41598-026-50900-9 PMC13365603 42129231

[64] Zhang YB, Xu L, Yuan M, Liu MF, Li Y, Zhong XL, Lin ZX, Xian YF, Lu P, Xi ZC, Xu HX (2026) Protective effects of *Prunella vulgaris* polysaccharides against herpes simplex virus type 1 infection through the STING-TBK1-IRF3 pathway. J Integr Med 24:454–465 10.1016/j.joim.2025.12.008 41506946

[65] Deng H, Jian Z, Zhu L, Li F, Zhao J, Deng J, Sun X, Xu Z (2022) Investigation of the anti-pseudorabies virus activity of interferon lambda 3 in cultured porcine kidney epithelial cells. Vet Med Sci 8:2444–2450 10.1002/vms3.933 PMC9677384 36122903

