# Supplementary material for: Exosome-mediated transfer of bta-miR-2484 upregulates JAK-STAT pathway to inhibit BoHV-1 replication by targeting SOCS5
Source: Vet Res. 2026 Nov 9;57:198. doi: 10.1186/s13567-026-01875-y (PMC13749771; doi:10.1186/s13567-026-01875-y)
Supplement: Supplementary file 1 [file 13567_2026_1875_MOESM1_ESM.docx]

Additional file 1. Sequences of miRNA mimics, inhibitors and siRNAs.

| miRNAs/siRNAs | Sequences (5’-3’) | |
| --- | --- | --- |
|  | sense (5’-3’) | antisense (5’-3’) |
| bta-miR-2484 mimics | GAGCUAUGAUGACUUUGAUUGCAU | GCAAUCAAAGUCAUCAUAGCUCUU |
| bta-miR-2484 inhibitor | AUGCAAUCAAAGUCAUCAUAGCUC |  |
| si SOCS5 | GGGUUAAGCCCUUCAAAGATT | UCUUUGAAGGGCUUAACCCTT |
| cel-miR-39 mimic | UCACCGGGUGUAAAUCAGCUUG |  |
