# Supplementary material for: Exosome-mediated transfer of bta-miR-2484 upregulates JAK-STAT pathway to inhibit BoHV-1 replication by targeting SOCS5
Source: Vet Res. 2026 Nov 9;57:198. doi: 10.1186/s13567-026-01875-y (PMC13749771; doi:10.1186/s13567-026-01875-y)
Supplement: Supplementary file 2 [file 13567_2026_1875_MOESM2_ESM.docx]

Additional file 2. Sequences of primers used in the research.

| Primers | Sequences (5’-3’) |
| --- | --- |
| Primers for CDS Cloning |  |
| SOCS5-CDS-F | CGCGGATCCGCCACCATGGATAAAGTGGGGAAAATGTGG |
| SOCS5-CDS-R | CCGCTCGAGTTACTTTGCTTTGACTGGTTCTCG |
| SOCS5 3’UTR-F | TCTAGTTGTTTAAACGAGCTCAGTTCTTAAAATTTCAGACTT |
| SOCS5 3’UTR-R | CAGGTCGACTCTAGACTCGAGTTTTCC |
| Primers for RT-qPCR |  |
| RT-cel-miR-39 | GTCGTATCCAGTGCAGGGTCCGAGGTATTCGCACTGGATACGACCAAGCT |
| cel-miR-39-F | GCCGCTCACCGGGTGTAAATC |
| cel-miR-39-R | GTGCAGGGTCCGAGGT |
| RT-U6 | AACGCTTCACGAATTTGCGT |
| U6-F | CTCGCTTCGGCAGCACA |
| U6-R | AACGCTTCACGAATTTGCGT |
| RT-bta-miR-2484 | GTCGTATCCAGTGCAGGGTCCGAGGTATTCGCACTGGATACGACATGCAA |
| bta-miR-2484-F | GCGGAGCTATGATGACTTTGA |
| bta-miR-2484-R | AGTGCAGGGTCCGAGGTATT |
| RT-bta-miR-2404 | GTCGTATCCAGTGCAGGGTCCGAGGTATTCGCACTGGATACGACGCAGAT |
| bta-miR-2404-F | CGCGTTGCACTGCATGGT |
| bta-miR-2404-R | AGTGCAGGGTCCGAGGTATT |
| RT-bta-miR-1434-5p | GTCGTATCCAGTGCAGGGTCCGAGGTATTCGCACTGGATACGACAGAAAT |
| bta-miR-1434-5p-F | GCGCGGTACATGATGACTAAA |
| bta-miR-1434-5p-R | AGTGCAGGGTCCGAGGTATT |
| RT-bta-miR-2463 | GTCGTATCCAGTGCAGGGTCCGAGGTATTCGCACTGGATACGACCAGACA |
| bta-miR-2463-F | CGCTGCATGTGGTGGAAA |
| bta-miR-2463-R | AGTGCAGGGTCCGAGGTATT |
| RT-bta-miR-491 | GTCGTATCCAGTGCAGGGTCCGAGGTATTCGCACTGGATACGACCCTCAT |
| bta-miR-491-F | CGAGTGGGGAACCCTTCC |
| bta-miR-491-R | AGTGCAGGGTCCGAGGTATT |
| RT-bta-miR-193a-3p | GTCGTATCCAGTGCAGGGTCCGAGGTATTCGCACTGGATACGACACTGGG |
| bta-miR-193a-3p-F | CGCGAACTGGCCTACAAAGT |
| bta-miR-193a-3p-R | AGTGCAGGGTCCGAGGTATT |
| RT-bta-miR-2411 | GTCGTATCCAGTGCAGGGTCCGAGGTATTCGCACTGGATACGACGGATGT |
| bta-miR-2411-F | CGGCTGAACTGTCTTACTCCC |
| bta-miR-2411-R | AGTGCAGGGTCCGAGGTATT |
| RT-bta-miR-1434-3p | GTCGTATCCAGTGCAGGGTCCGAGGTATTCGCACTGGATACGACCCTCAG |
| bta-miR-1434-3p-F | GCGCGGAAGAAATCTAAGGT |
| bta-miR-1434-3p-R | AGTGCAGGGTCCGAGGTATT |
| RT-bta-miR-433 | GTCGTATCCAGTGCAGGGTCCGAGGTATTCGCACTGGATACGACACACCG |
| bta-miR-433-F | GCGATCATGATGGGCTCCT |
| bta-miR-433-R | AGTGCAGGGTCCGAGGTATT |
| RT-bta-miR-543 | GTCGTATCCAGTGCAGGGTCCGAGGTATTCGCACTGGATACGACAAGAAG |
| bta-miR-543-F | CGAAACATTCGCGGTGCA |
| bta-miR-543-R | AGTGCAGGGTCCGAGGTATT |
| RT-bta-miR-2388 | GTCGTATCCAGTGCAGGGTCCGAGGTATTCGCACTGGATACGACTCAGCA |
| bta-miR-2388-F | CGAGCTCCCGTCTCCTCTG |
| bta-miR-2388-R | AGTGCAGGGTCCGAGGTATT |
| RT-bta-miR-99a-5p | GTCGTATCCAGTGCAGGGTCCGAGGTATTCGCACTGGATACGACACAAGA |
| bta-miR-99a-5p-F | GCGAACCCGTAGATCCGA |
| bta-miR-99a-5p-R | AGTGCAGGGTCCGAGGTATT |
| RT-bta-miR-193a-5 | GTCGTATCCAGTGCAGGGTCCGAGGTATTCGCACTGGATACGACTCATCT |
| bta-miR-193a-5-F | TGGGTCTTTGCGGGCG |
| bta-miR-193a-5-R | AGTGCAGGGTCCGAGGTATT |
| RT-bta-miR-199a-3P | GTCGTATCCAGTGCAGGGTCCGAGGTATTCGCACTGGATACGACTAACCA |
| bta-miR-199a-3p-F | GCGCGACAGTAGTCTGCACAT |
| bta-miR-199a-3p-R | AGTGCAGGGTCCGAGGTATT |
| SOCS5-F | ATGGGGACAGTTGTGCAGTT |
| SOCS5-R | TCAATCTGCGTGTGGACTTT |
| BHV-1 gE-F | GCCAGTTAGCGACGACGAAT |
| BHV-1 gE-R | ACTTTGAACCCAGAGCGACT |
| ISG15-F | GGAGGCCCATGGATGATG |
| ISG15-R | CCGAAGACGTAGATTCATGAACAC |
| OAS1-F | GACGTGCTTCCAAGAGTCCA |
| OAS1-R | GGAAGACGACAAGGTCAGCA |
| β-actin-F | GATGAGATTGGCATGGCTTTA |
| β-actin-R | AACCGACTGCTGTCACCTTC |
