# Supplementary material for: Exosome-mediated transfer of bta-miR-2484 upregulates JAK-STAT pathway to inhibit BoHV-1 replication by targeting SOCS5
Source: Vet Res. 2026 Nov 9;57:198. doi: 10.1186/s13567-026-01875-y (PMC13749771; doi:10.1186/s13567-026-01875-y)
Supplement: Supplementary file 3 [file 13567_2026_1875_MOESM3_ESM.docx]

Additional file 3. Evaluation of viral contamination in the purified exosome preparations. (A) Morphological observation of cytopathic effects (CPE) in MDBK cells treated with exosomes derived from different groups. (B) BoHV-1 gE copy numbers in MDBK cells at 72 h post-inoculation with exosomes from different groups.
