# Supplementary material for: Exosome-mediated transfer of bta-miR-2484 upregulates JAK-STAT pathway to inhibit BoHV-1 replication by targeting SOCS5
Source: Vet Res. 2026 Nov 9;57:198. doi: 10.1186/s13567-026-01875-y (PMC13749771; doi:10.1186/s13567-026-01875-y)
Supplement: Supplementary file 4 [file 13567_2026_1875_MOESM4_ESM.docx]

Additional file 4. Summary of all differentially expressed miRNAs.

| Gene | Log_2_FoldChange | pval | padj | Up/Down | Significant |
| --- | --- | --- | --- | --- | --- |
| bta-miR-2404 | 4.976039258 | 2.14E-15 | 1.28E-14 | up | yes |
| bta-miR-2484 | 3.697832247 | 2.46E-74 | 4.57E-73 | up | yes |
| bta-miR-1434-5p | 3.610457116 | 0.003091 | 0.007851 | up | yes |
| bta-miR-2463 | 3.255973343 | 1.19E-09 | 5.95E-09 | up | yes |
| bta-miR-6122-3p | 3.230752786 | 0.011856 | 0.025978 | up | yes |
| bta-miR-491 | 3.152639663 | 5.53E-50 | 8.53E-49 | up | yes |
| bta-miR-193a-3p | 3.002156232 | 8.85E-50 | 1.32E-48 | up | yes |
| bta-miR-2411-3p | 2.757568524 | 3.10E-85 | 7.69E-84 | up | yes |
| bta-miR-1434-3p | 2.630972933 | 2.21E-07 | 9.16E-07 | up | yes |
| bta-miR-374b | 2.615607578 | 1.16E-84 | 2.74E-83 | up | yes |
| bta-miR-2427 | 2.512262142 | 0.00046 | 0.001318 | up | yes |
| bta-miR-19b | 2.477259073 | 5.19E-109 | 1.66E-107 | up | yes |
| bta-miR-19a | 2.235365095 | 6.76E-41 | 8.63E-40 | up | yes |
| bta-miR-582 | 2.201140265 | 0.012755 | 0.027812 | up | yes |
| bta-miR-331-3p | 2.046148679 | 5.31E-86 | 1.40E-84 | up | yes |
| bta-miR-29c | 1.923013088 | 6.82E-49 | 9.84E-48 | up | yes |
| bta-miR-500 | 1.918624952 | 4.17E-05 | 0.000137 | up | yes |
| bta-miR-29d-5p | 1.896844029 | 5.96E-07 | 2.36E-06 | up | yes |
| bta-miR-129-3p | 1.889405728 | 1.03E-19 | 7.32E-19 | up | yes |
| bta-miR-424-5p | 1.812981386 | 3.78E-51 | 6.04E-50 | up | yes |
| bta-miR-138 | 1.691299409 | 0.003937 | 0.00983 | up | yes |
| bta-miR-106a | 1.658364923 | 0.002658 | 0.006867 | up | yes |
| bta-miR-195 | 1.646665678 | 4.99E-06 | 1.84E-05 | up | yes |
| bta-miR-16b | 1.64420707 | 9.91E-37 | 1.17E-35 | up | yes |
| bta-miR-1388-3p | 1.578627535 | 0.000756 | 0.002111 | up | yes |
| bta-miR-190a | 1.577073949 | 0.00224 | 0.005822 | up | yes |
| bta-miR-324 | 1.53060202 | 6.59E-06 | 2.36E-05 | up | yes |
| bta-miR-30b-5p | 1.527515135 | 4.69E-191 | 2.62E-189 | up | yes |
| bta-miR-1388-5p | 1.450938218 | 0.005591 | 0.013582 | up | yes |
| bta-miR-101 | 1.44873935 | 9.30E-202 | 5.94E-200 | up | yes |
| bta-miR-23b-3p | 1.406976366 | 4.01E-180 | 1.63E-178 | up | yes |
| bta-miR-23a | 1.400270754 | 4.53E-224 | 4.05E-222 | up | yes |
| bta-miR-18a | 1.391427956 | 2.69E-15 | 1.58E-14 | up | yes |
| bta-miR-20a | 1.357978913 | 7.79E-101 | 2.32E-99 | up | yes |
| bta-miR-29e | 1.326591012 | 0.009249 | 0.021094 | up | yes |
| bta-miR-2424 | 1.279004878 | 0.023828 | 0.049084 | up | yes |
| bta-miR-34a | 1.266189633 | 2.58E-79 | 5.24E-78 | up | yes |
| bta-miR-452 | 1.260261464 | 2.11E-30 | 2.05E-29 | up | yes |
| bta-miR-708 | 1.199745468 | 0.006381 | 0.015173 | up | yes |
| bta-miR-130a | 1.112069566 | 1.36E-10 | 7.09E-10 | up | yes |
| bta-miR-503-5p | 1.099608327 | 6.05E-28 | 5.31E-27 | up | yes |
| bta-miR-301a | 1.09817312 | 0.00878 | 0.020231 | up | yes |
| bta-miR-9-3p | 1.062461233 | 3.44E-06 | 1.28E-05 | up | yes |
| bta-miR-130b | 1.058488141 | 0.000432 | 0.001255 | up | yes |
| bta-miR-2285ab | 1.057302423 | 2.89E-05 | 9.58E-05 | up | yes |
| bta-miR-210 | 1.056355859 | 6.92E-76 | 1.34E-74 | up | yes |
| bta-miR-29b | 1.049574863 | 9.08E-07 | 3.53E-06 | up | yes |
| bta-miR-2440 | 1.043627689 | 1.02E-22 | 7.75E-22 | up | yes |
| bta-miR-17-3p | 1.001859406 | 2.74E-07 | 1.11E-06 | up | yes |
| bta-miR-885 | -1.026358943 | 1.05E-13 | 6.07E-13 | down | yes |
| bta-miR-200b | -1.03446631 | 6.57E-06 | 2.36E-05 | down | yes |
| bta-miR-574 | -1.107607147 | 1.69E-25 | 1.42E-24 | down | yes |
| bta-miR-92a | -1.125884328 | 3.32E-203 | 2.47E-201 | down | yes |
| bta-let-7a-5p | -1.228049938 | 8.37E-181 | 3.74E-179 | down | yes |
| bta-miR-128 | -1.237051054 | 1.89E-36 | 2.16E-35 | down | yes |
| bta-miR-139 | -1.323506882 | 0.002908 | 0.007471 | down | yes |
| bta-miR-2320-3p | -1.329458307 | 0.007541 | 0.017557 | down | yes |
| bta-miR-1307 | -1.341497308 | 4.45E-81 | 9.48E-80 | down | yes |
| bta-miR-365-5p | -1.376978464 | 3.20E-09 | 1.54E-08 | down | yes |
| bta-miR-505 | -1.44392622 | 5.80E-19 | 3.99E-18 | down | yes |
| bta-miR-27a-5p | -1.445796483 | 1.58E-23 | 1.22E-22 | down | yes |
| bta-miR-760-3p | -1.506731715 | 0.00051 | 0.001451 | down | yes |
| bta-miR-142-5p | -1.557914056 | 0.015661 | 0.033669 | down | yes |
| bta-miR-6517 | -1.564701336 | 8.03E-06 | 2.83E-05 | down | yes |
| bta-let-7c | -1.60713009 | 1.17E-19 | 8.20E-19 | down | yes |
| bta-miR-4449 | -1.655548932 | 5.60E-05 | 0.00018 | down | yes |
| bta-miR-451 | -1.718450388 | 0.011805 | 0.025978 | down | yes |
| bta-miR-1246 | -1.752442986 | 0 | 0 | down | yes |
| bta-miR-2904 | -1.782671356 | 7.95E-09 | 3.66E-08 | down | yes |
| bta-miR-320a | -1.793411509 | 3.82E-143 | 1.42E-141 | down | yes |
| bta-miR-2447 | -1.80697274 | 0.001485 | 0.003999 | down | yes |
| bta-miR-2448-3p | -1.841408234 | 0.000125 | 0.000394 | down | yes |
| bta-miR-205 | -1.85178195 | 0.018924 | 0.039901 | down | yes |
| bta-miR-2443 | -1.860707396 | 2.06E-17 | 1.35E-16 | down | yes |
| bta-miR-125a | -1.878016271 | 1.05E-98 | 2.93E-97 | down | yes |
| bta-let-7d | -1.947802693 | 1.57E-137 | 5.39E-136 | down | yes |
| bta-miR-424-3p | -1.966170532 | 8.31E-49 | 1.16E-47 | down | yes |
| bta-miR-2431-3p | -2.017901253 | 1.09E-29 | 9.97E-29 | down | yes |
| bta-miR-503-3p | -2.027424695 | 0.009247 | 0.021094 | down | yes |
| bta-miR-423-5p | -2.043469212 | 0 | 0 | down | yes |
| bta-miR-486 | -2.224256416 | 5.86E-30 | 5.45E-29 | down | yes |
| bta-miR-1260b | -2.247595095 | 2.94E-18 | 1.99E-17 | down | yes |
| bta-miR-2382-5p | -2.250807723 | 0.005937 | 0.014269 | down | yes |
| bta-miR-219 | -2.330260407 | 0.013138 | 0.028507 | down | yes |
| bta-miR-2340 | -2.364559505 | 0.023255 | 0.048125 | down | yes |
| bta-miR-122 | -2.373349589 | 3.83E-09 | 1.82E-08 | down | yes |
| bta-let-7b | -2.380658936 | 0 | 0 | down | yes |
| bta-miR-2474 | -2.605331136 | 2.92E-06 | 1.10E-05 | down | yes |
| bta-miR-615 | -2.67957797 | 0.006951 | 0.016354 | down | yes |
| bta-miR-126-5p | -2.944030329 | 0.001526 | 0.004086 | down | yes |
| bta-miR-379 | -2.967820377 | 0.000321 | 0.000944 | down | yes |
| bta-miR-150 | -3.164883008 | 0.000175 | 0.000536 | down | yes |
| bta-miR-2478 | -3.174695182 | 2.01E-32 | 2.09E-31 | down | yes |
| bta-miR-199a-5p | -3.283320853 | 2.38E-39 | 2.95E-38 | down | yes |
| bta-miR-23b-5p | -3.395558463 | 1.14E-11 | 6.16E-11 | down | yes |
| bta-miR-411c-5p | -3.444288998 | 0.0083 | 0.019223 | down | yes |
| bta-miR-143 | -3.475468018 | 4.86E-18 | 3.24E-17 | down | yes |
| bta-miR-380-3p | -3.478748747 | 0.000122 | 0.000387 | down | yes |
| bta-miR-494 | -3.507855757 | 2.15E-07 | 9.05E-07 | down | yes |
| bta-miR-126-3p | -3.581767598 | 2.74E-17 | 1.75E-16 | down | yes |
| bta-miR-199a-3p | -3.603816372 | 2.47E-84 | 5.52E-83 | down | yes |
| bta-miR-432 | -3.81373488 | 0.002093 | 0.00547 | down | yes |
| bta-miR-154c | -3.82366475 | 0.001989 | 0.00523 | down | yes |
| bta-miR-409a | -3.919189755 | 0.022153 | 0.046058 | down | yes |
| bta-miR-193a-5p | -3.97657631 | 0 | 0 | down | yes |
| bta-miR-382 | -4.380636793 | 0.000218 | 0.000662 | down | yes |
| bta-miR-411a | -4.428900505 | 1.95E-09 | 9.57E-09 | down | yes |
| bta-miR-483 | -4.601168565 | 1.35E-05 | 4.62E-05 | down | yes |
| bta-miR-99a-5p | -4.754698023 | 3.12E-36 | 3.49E-35 | down | yes |
| bta-miR-410 | -4.80179674 | 0.005729 | 0.013843 | down | yes |
| bta-miR-127 | -4.962336765 | 5.58E-17 | 3.47E-16 | down | yes |
| bta-miR-199b | -5.223276448 | 1.25E-24 | 9.96E-24 | down | yes |
| bta-miR-363 | -5.235179562 | 0.010238 | 0.023112 | down | yes |
| bta-miR-2388-5p | -5.364474027 | 0.015667 | 0.033669 | down | yes |
| bta-miR-543 | -5.793865002 | 0.001233 | 0.003362 | down | yes |
| bta-miR-433 | -6.442603156 | 4.81E-05 | 0.000156 | down | yes |
